# Quantification of Biodriven Transfer of Per- and Polyfluoroalkyl Substances from the Aquatic to the Terrestrial Environment via Emergent Insects

**DOI:** 10.1021/acs.est.0c07129

**Published:** 2021-05-24

**Authors:** Alina Koch, Micael Jonsson, Leo W. Y. Yeung, Anna Kärrman, Lutz Ahrens, Alf Ekblad, Thanh Wang

**Affiliations:** † Man-Technology-Environment Research Centre, Örebro University, 70182 Örebro, Sweden; ‡ Department of Ecology and Environmental Sciences, Umeå University, 90187 Umeå, Sweden; § Department of Aquatic Sciences and Assessment, Swedish University of Agricultural Sciences (SLU), 75007 Uppsala, Sweden

**Keywords:** PFAS, biodriven transfer, emergent aquatic insects, terrestrial consumers

## Abstract

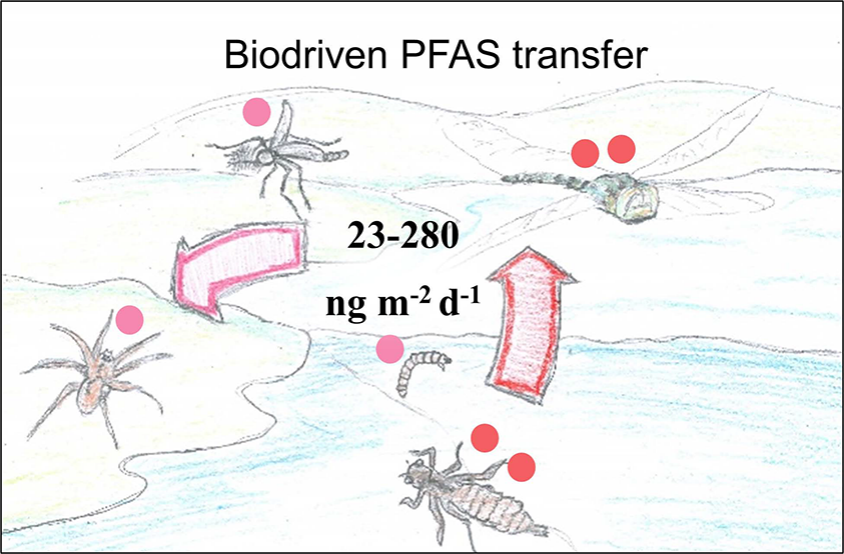

Emergent aquatic insects are important food subsidies to riparian food webs but can also transfer waterborne contaminants to the terrestrial environment. This study aimed to quantitatively assess this biodriven transfer for per- and polyfluoroalkyl substances (PFAS). Aquatic insect larvae, emergent aquatic insects, terrestrial consumers, sediment, and water were collected from a contaminated lake and stream and an uncontaminated pond, and analyzed for PFAS and stable isotopes of carbon and nitrogen. Top predators in this study were spiders, which showed the highest average ∑_24_PFAS concentration of 1400 ± 80 ng g^–1^ dry weight (dw) at the lake and 630 ng g^–1^ dw at the stream. The transfer of PFAS from the lake to the riparian zone, via deposition of emergent aquatic insects, was 280 ng ∑_24_PFAS m^–2^ d^–1^ in 2017 and only 23 ng ∑_24_PFAS m^–2^ d^–1^ in 2018. Because of higher production of emergent aquatic insects, the lake had higher PFAS transfer and higher concentrations in terrestrial consumers compared to the stream, despite the stream having higher PFAS concentration in water and aquatic insect larvae. Our results indicate that biodriven transfer of PFAS from the aquatic systems and subsequent uptake in terrestrial food webs depend more on emergence amounts, i.e., aquatic prey availability, rather than on PFAS concentrations in water and aquatic prey.

## Abstract 2

PFAS are transferred from contaminated aquatic to the terrestrial ecosystems via insect emergence and the quantity can be substantial compared to other local depositions of PFAS.

## Introduction

Per- and polyfluoroalkyl substances (PFAS) are contaminants of emerging concern and are widely used in the industry and in consumer products because of their unique physicochemical properties (e.g., as surfactants). PFAS can be very resistant to degradation, can cause a range of adverse health effects, and can be highly bioaccumulative depending on their fluorocarbon chain length and functional group.^1^ A subgroup of PFAS, perfluoroalkyl acids (PFAA), has been of high environmental concern due to high persistency. PFAA are ubiquitously found in the environment, and >95% of their emissions have been estimated to be released into aquatic environments.^2,3^ Once in the aquatic ecosystem, many PFAS can easily distribute and bioaccumulate in the aquatic food webs.^4,5^

Aquatic ecosystems interact with the surrounding terrestrial environment via transfer of energy and matter, primarily via the movement of emergent aquatic organisms.^6^ These transfers are important ecosystem functions. For example, emergent aquatic insects are essential food subsidies to bats, reptiles, amphibians, spiders, and birds at the riparian zone. Aquatic insect emergence varies seasonally and geographically, and can range from hundreds to 150,000 individuals per square meter per year (as reviewed by Jackson and Fisher^7^). Emergence from central Swedish wetlands is known to range from 1200 to 4300 individuals per square meter per year,^8^ or in terms of biomass (dry weight) 0.5–2.5 grams per square meter per year from Finnish lentic systems.^9^ Differences in insect emergence among aquatic ecosystems are primarily driven by variation in secondary production, aquatic invertebrate community composition, and ecosystem geometry.^9,10^ Once emerged, most insects deposit on land directly adjacent to the aquatic origin (i.e., riparian zone), though some taxa (e.g., dragonflies) can disperse over long distances.^11^ The response of riparian consumers, such as spiders, to high aquatic insect emergence can be substantial in terms of their density and relative proportion of aquatic prey in their diet.^12,13^

Aquatic prey subsidies are not necessarily only beneficial to terrestrial consumers, as they also can contain contaminants that the aquatic insects are exposed to in water and sediment before emerging as adults. Hence, contaminants that bioaccumulate internally can be moved from water to land at emergence. Such biodriven transfer has been described for nitrogen,^14^ heavy metals,^15−17^ algal toxins,^18^ pharmaceuticals,^19^ and lipophilic persistent organic pollutants such as polybrominated diphenyl ethers (PBDEs),^20^ polychlorinated biphenyls (PCBs),^21−25^ and 2,3,7,8-tetrachlorodibenzofuran.^26^ Spiders as well as mayflies have been used as biomonitoring species for movements of contaminants,^22,27^ but environmental consequences of such movements have rarely been investigated, although they may influence community structure and health indicators of terrestrial insectivores.^28,29^

**Figure 1:**
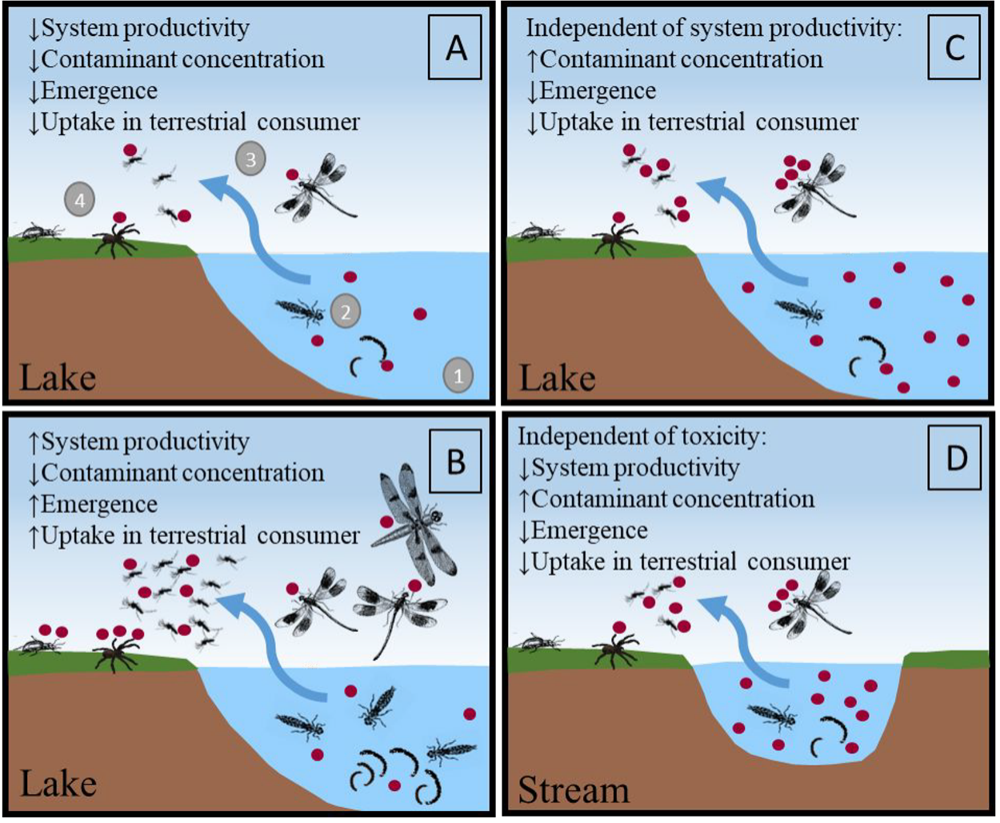
Conceptual figure of potential scenarios (plots A–D) of contaminant transfer from water to land in a lake (A, B, and C) and a stream (D). Numbers 1–4 in plot A represent factors that are important for the transfer: (1) contaminant concentration in the surface water, (2) uptake by aquatic insects, (3) transfer of the contaminant via insect emergence, and (4) consumption by riparian consumers. Small arrows indicate status of the system, where contaminant concentration refers to PFAS concentration in surface water, and scenario D illustrates a situation where toxicity has no effect on emergence.

There are several factors that can influence biodriven transfer of contaminants, such as PFAS, from water to land: (1) the concentration of the contaminant in the surface water/sediment and its bioavailability and toxicity, (2) level of uptake in aquatic insects (i.e., bioconcentration and biomagnification), (3) quantity of emergent aquatic insect biomass moving from water to land (i.e., the productivity of the aquatic system), and (4) diet of terrestrial consumers (i.e., availability of aquatic prey relative to terrestrial prey).^16^ Based on this, we identified four possible scenarios of biodriven contaminant transfer (Figure 1): (A) a low-production aquatic system with low contaminant concentration in the water, resulting in low emergence and, thus, low uptake by terrestrial consumers; (B) a highly productive aquatic system with low contaminant concentrations in the water, resulting in high emergence and, thus, comparatively high uptake by riparian consumers. This high uptake at high emergence can arise from terrestrial consumers switching to aquatic prey exclusively when their availability is high.^12,13^ Hence, in this scenario, terrestrial consumers show unexpectedly high internal contaminant concentrations, in relation to the contaminant concentration in aquatic prey. A third scenario (C) is when the contaminant concentration has reached a toxic threshold, reducing the emergence of aquatic insects and, thus, the uptake by terrestrial consumers.^16,28^ This is independent of the potential productivity of the aquatic system. Lastly, in a fourth scenario (D), the aquatic system is simply not productive enough (e.g., small streams) to produce sufficient amounts emergent aquatic prey that results in a noticeable uptake in terrestrial consumers. This is independent of the internal concentration of each aquatic prey. As such, it should be important to identify which factors drive the contaminant transfer and uptake in terrestrial food webs, to obtain a mechanistic understanding which can enable more efficient remediation actions.

Aquatic organisms can take up and bioaccumulate some PFAS.^30−32^ In a previous study, we investigated a pond contaminated by aqueous film forming foam (AFFF), and found high concentrations of 24 PFAS in aquatic invertebrates, including emergent aquatic insects, such as adult dragonflies.^33^ Hence, terrestrial consumers feeding on emergent aquatic insects from PFAS-contaminated systems could be exposed to PFAS via predation.^33^ However, the biomass of emergent aquatic insects collected in that study was not sufficient for quantification of PFAS transferred from water to land.^33^ Here, we extend this concept by providing quantitative data on PFAS and explore biodriven transport based on different scenarios for three other sites of varying size, productivity, and contamination extent. To do this, we collected a comprehensive set of samples in 2017 and in 2018, in a PFAS-affected area in Sweden. Samples included aquatic insect larvae, emergent aquatic insects, terrestrial consumers, sediment, and water, at two sites near a PFAS point source, as well as at a reference site. Chemical analysis included 26 target PFAS and branched isomers of PFOS and PFHxS, as well stable isotope analysis of carbon and nitrogen to elucidate food web structure and nutrient sources. The aim was to provide quantitative estimates of PFAS transfer from water to land (i.e., area-based deposition on land via emergent aquatic insects) and to measure their uptake in terrestrial consumers. To obtain an understanding of which factors regulate the transfer, we conceptualize and discuss the results in relation to the different scenarios presented in Figure 1.

## Sampling and Experimental Section

### Study Site

**Figure 2:**
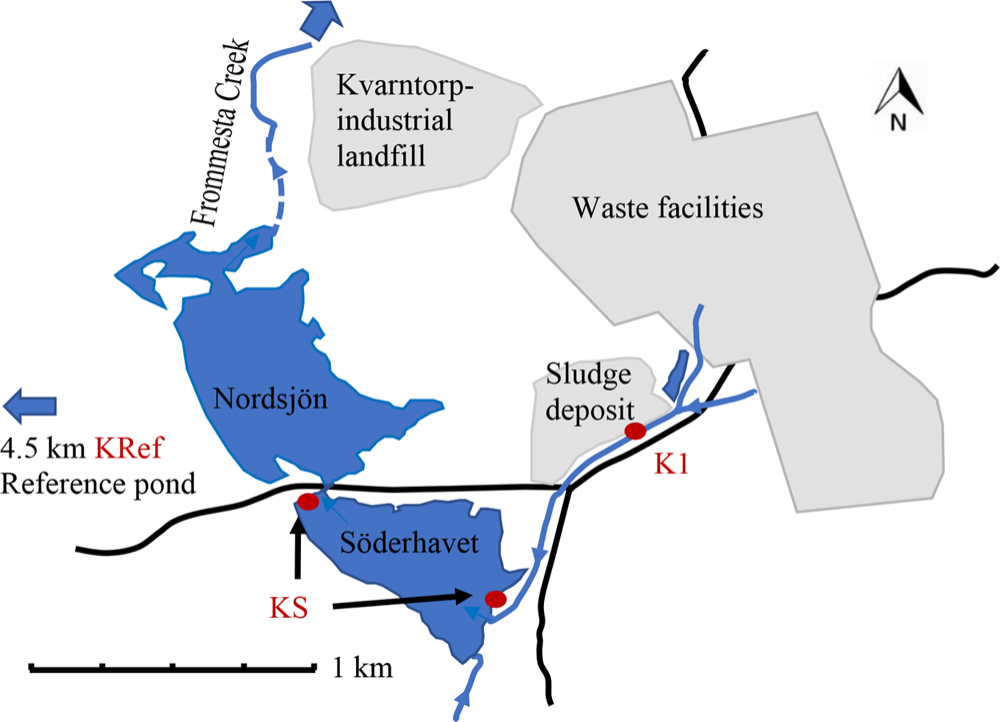
Map of sampling area at Kvarntorp, Kumla municipality, Sweden, with the sampling sites K1 (the stream), KS (two sites at the lake Söderhavet), and KRef (the reference pond).

Kvarntorp is an industrial area which is part of the municipality of Kumla, located in central Sweden (Figure 2). The area has historically been impacted by heavy industry, and nowadays Sweden’s only hazardous waste management facility is loacted in this area. In 2015, PFAS contamination in fish from lake Söderhavet was discovered, with an average PFOS concentration of 750 ng g^–1^ wet weight (ww).^34^ The lake has an area of ca. 29 ha and receives about 4.7 million m^3^ of water per year from the stream (Ulfstorpsbäcken) in the Kvarntorp area.^35^ Sources of PFAS at Kvarntorp are most likely complex due to the different industrial activities. More information about the industrial history and PFAS concentration measured previously can be found under “Study site” in the Supporting Information (SI).

For this study, samples were collected from the stream Ulfstorpsbäcken (K1, referred to as “the stream” in the discussion, 59°6′53.58″N, 15°16′8.90″E) and two sites at Söderhavet (59°6′45.03″N, 15°14′54.02″E): one located close to the inflow and one close to the outflow of the lake (Figure 2). Samples taken at Söderhavet were combined from both sites, and they are referred to as KS or “the lake”. A pond located 4.5 km west was chosen as a reference site (KRef, referred to as “the reference pond”, 59°6′52.02″N, 15°10′25.87″E).

### Sampling Campaigns

Various matrices were collected from all three sites in 2017 (May 31 to June 15; October 16–17) and 2018 (May 2 to July 3). Grab samples of surface water were taken in spring and fall 2017 as well as in spring 2018. Polypropylene (PP) bottles were rinsed three times with surface water and then filled from >10 cm below the surface and >1 m away from the shore. Water chemistry parameters (pH, alkalinity, water temperature) were determined during water sampling, and water samples collected in fall 2017 were analyzed for 19 water chemical parameters (Table S1 in Supporting Information (SI)) at the Department of Soil and Environment at the Swedish University of Agricultural Science. Several sediment cores with a maximum depth of 20 cm were sampled with a core borer (diameter 5 cm) in October 2017 and were combined as composite samples resulting in one sample per site. During the soil sampling, earthworms were collected from the surface soil (<20 cm depth), rinsed with Milli-Q-water, and kept in a plastic container on wet filter (prebaked glass microfiber filters [GFF] filter; 450 °C for 3 h) for 48 h to clean their guts, before being frozen. All worms were combined to a composite sample per site sampled.

Deposition traps (18 × 18 × 9 cm^3^ squared plastic boxes), to collect aquatic emergent insects, were deployed for a period of 16 days in spring 2017 (May 31 to June 15).^29,36^ Sets of three deposition traps were placed about 5 m from each other within 5 m from the shore at three locations per sampling site, resulting in nine traps per site (double at the lake since two sampling locations were there). In 2018, deposition traps were deployed during the entire early spring emergence (May 2 to July 3). During that period, deposition traps were collected and refilled four times; after the first 13 days (T1), after another 13 days (T2), thereafter after 16 days (T3), and after 21 days (T4). In 2018, sets of two deposition traps were placed at three locations per sampling site, resulting in six traps per site. Terrestrial consumers were collected in pitfall traps during spring 2017. The pitfall traps were 250 mL PPcups buried with the rim of the cup at ground level. Each pitfall trap was placed a few centimeters from a deposition trap, resulting in nine traps per site. In all deposition and pitfall traps, a mixture of 50% propylene glycol, 50% Milli-Q water, and a few drops of detergent to break the surface tension was used as a preservative. Glycol from a few traps was extracted to test for PFAS contamination, and the test revealed that no PFAS were above the detection limits. Samples from all the traps were stored at 4 °C until they were sorted into selected taxonomic groups, pooled, weighed, and then frozen. Before chemical analysis, each sample was freeze-dried and the dry weight determined.

Flying emerging insects were captured with a sweep net (up to 10 m from the shore) during sampling periods T1 and T2 in 2018, and these were analyzed for both PFAS and stable isotopes. The reason only sweep net samples were used for isotope analysis was because insects from deposition traps were soaked in glycol, and potential influence from carbons in glycol on the isotope ratios could not be ruled out (see section “Stable isotope analysis” in the Supporting Information). Additionally, aquatic invertebrates were collected with a kick net (dimension 23 cm diameter, mesh size 500 μm, 1.5 m handle) in fall 2017. In brief, the net was moved with fast turns for 1 min at the shoreline to stir up bottom sediment and to collect the released suspended material and aquatic invertebrates. Kick-net samples were collected at the shoreline close to where the deposition traps were placed during spring. Therefore, three locations were sampled three times, resulting in nine samples per site. The insects from these samples were most likely to emerge in spring 2018 and, thus, potentially those ending up as prey in the terrestrial food web. All samples from the sweep net and kick-net sampling were frozen until they were sorted and pooled in the lab for chemical analysis.

A total of 108 samples were analyzed for PFAS. Sample groups were as follows: water, sediment, aquatic insect larvae, emergent aquatic insects, terrestrial consumers, earthworms, and plants. Sample grouping criteria were as follows: samples of invertebrate groups were pooled per year, site, and sampling method (i.e., deposition trap samples were kept separate from sweep net samples). The group aquatic insect larvae consisted of all insect larvae that were caught, mainly dragonfly, damselfly, and alderfly larvae. Emergent aquatic insects represent the adult emergent insects captured by deposition traps and sweep nets and were midges, dragonflies, and damselflies. Terrestrial consumers in this study included a variety of riparian invertebrates, and were mainly spiders, beetles, and ants. For pitfall and deposition trap collections, all nine replicates per site were pooled to obtain sufficient biomass for later analysis. In cases where more than sufficient biomass was collected, duplicates or triplicates for a sample group were used. Detailed information about each sample can be seen in Table S2 in Supporting Information.

Deposition of emergent aquatic insects was collected within 3 m from the lake/stream shore and this distance was therefore defined as the riparian zone surrounding the aquatic systems in this area. Estimation of emergence, number of insects, and dry biomass, as well as PFAS transfer, was based on emergent aquatic insects collected in the deposition traps at the riparian zone. Emergence was calculated by the sum of number of insects or weight of dry biomass from the whole period divided by the number of traps used to collect these and corrected by the trap area to get square meters. Finally, the value was divided by the number of days to get the mean number/biomass of insects deposited onto land for each of the sites. PFAS transfer was estimated by the value of emerged biomass (g dw m^–2^ d^–1^) multiplied by ∑_24_PFAS. To estimate PFAS transfer for the whole riparian zone of Söderhavet, the estimated area of the 3-m-wide riparian zone surrounding the lake (the circular ring area: 5800 m^2^) was multiplied by the nanograms of PFAS determined for the specific sampling periods in 2017 (16 days) and 2018 (63 days). For the stream site, the sampling section of 50 m length and the riparian zone width of 2 × 3 m^2^ (to cover for both sides of the stream) were used to estimate transfer.

### Chemical Analysis

A total of 26 target PFAS were studied; PFCA with perfluorocarbon chain length of C_3_–C_13_, C_15_, and C_17_ (PFBA, PFPeA, PFHxA, PFHpA, PFOA, PFNA, PFDA, PFUnDA, PFDoDA, PFTrDA, PFTeDA, PFHxDA, PFOcDA), PFSA with perfluorocarbon chain length of C_4_–C_10_ and C_12_ (PFBS, PFPeS, PFHxS, PFHpS, PFOS, PFNS, PFDS, PFDoDS), fluorotelomer sulfonic acids (4:2, 6:2, 8:2, and 10:2 FTSA), and perfluorooctane sulfonamide (FOSA). More details about the target PFAS and addressed isotopically labeled PFAS for quantification can be found in Table S3 in Supporting Information. For the analysis of branched PFOS (br-PFOS) and PFHxS (br-PFHxS) isomers, two reference standards were used. The first one contained linear PFOS, 1m-PFOS, 6/2m-PFOS, 3/4/5m-PFOS, and 4.4/4.5/5.5-m2-PFOS (brPFOSK0113) and the second one contained linear PFHxS, 1m-PFHxS, 2/4m-PFHxS, 3m-PFHxS, and 3–3-m2-PFHxS (brPFHxSK0612). All reference PFAS were purchased from Wellington Laboratories (Guelph, Canada).

Description of water and solid sample extractions, chemical analysis of PFAS, and stable isotope analysis can be found in the SI. For isotope analysis, subsamples from the pooled invertebrate samples were used, if those had sufficient biomass for both PFAS and isotope analyses.

### Quality Control

During each batch extraction, two solvent blanks as well as one spiked (1 ng native standard) quality control (QC) sample were processed. The QC samples were Milli-Q-water for water and commercially purchased aquatic bloodworms (Chironomidae tetans) for the biota extractions (Table S4 in Supporting Information). Procedural blanks were in general <5% of the detected concentrations, and thus, the PFAS concentrations were not blank subtracted. Method detection limits (MDLs) were calculated for each batch as the average concentration of the solvent blanks plus three times the standard deviation (SD, Table S5 in Supporting Information). Method quantification limits (MQLs) were calculated by MDL × 3.3 (Table S5 in Supporting Information). For compounds where no peak was detected in the solvent blanks, the lowest point of calibration (20 pg) was used to calculate the detection limits. For calculations and graphs, values that were below the MQL but above the MDL were used without modification, whereas values below MDL were replaced by MDL/2. A detailed description of PFAS quantification can be found in the “Data analysis” section in the Supporting Information. Results were reported as concentrations on a dry weight basis (Tables S7 and S8 in Supporting Information). However, for comparison with other studies, concentrations were also converted to wet weight, using water contents determined for all invertebrate groups (Table S2 in Supporting Information).

During statistical comparisons, a PFAS was excluded when more than 50% of the samples showed values below MDL and groups with low number of samples (*n* ≤ 2) were excluded, as they cannot be statistically compared to the other groups. For pairwise comparison of PFAS concentration between invertebrate groups (i.e., aquatic insect larvae, emergent aquatic insects, and terrestrial consumers), the nonparametric Kruskal–Wallis was used with Dunn’s post hoc test, performed in R (version 3.6.0). Here, different taxa were pooled to obtain sufficient amounts for analysis, but we did not account for potential differences in the relative abundance of different taxa among samples and between years. Regression analysis was used to investigate the relationship between PFOS concentrations and δ^15^N in emergent aquatic insects and terrestrial invertebrate consumers.

## Results and Discussion

### PFAS Concentrations and Stable Isotope Results

This section discusses PFAS concentration in surface water, sediment, aquatic insects, emergent aquatic insects, and terrestrial consumers, as well as stable isotope ratio of carbon and nitrogen from all three sampling sites (the stream, the lake, and the reference pond). Sample information can be found in Table S2 in Supporting Information such as sample group, taxa, and number of individuals (quantity). PFAS were detected in all samples. Among the 26 PFAS, four compounds (i.e., PFTDA, PFHxDA, PFOcDA, and 4:2 FTSA) were below MDL in all samples. Furthermore, PFBA and PFPeA were excluded from this study, because their quantification could not be confirmed in most samples due to matrix effects.

#### Surface Water

Concentrations of ∑_24_PFAS in the stream were 700 ng L^–1^ in spring 2017, 2400 ng L^–1^ in fall 2017, and 1600 ng L^–1^ in spring 2018 (Figure S1 and Tables S7 and S8 in Supporting Information), whereas lake PFAS concentrations were about one order of magnitude lower at 370, 890, and 180 ng L^–1^ for the same sampling occasions, respectively, most likely due to dilution effect in the lake. Although three measurements over time are too few to draw any general conclusions, the observed temporal variation was likely due to seasonal differences in water discharge, i.e., the amount of water diluting the PFAS, and between-year variation in precipitation and temperature. PFAS concentration in the reference pond was <3 ng L^–1^, which is in accordance with environmental background concentrations for Swedish surface water.^37^ PFAS profiles in all water samples showed that PFOS was the most prominent PFAS (48–65% of total PFAS) followed by PFHxS and PFBS (Figure S2 in Supporting Information). PFAS profile patterns were similar for samples from the stream and the lake, indicating that the stream was the major point source of PFAS to Lake Söderhavet. In general, PFAS concentrations in the study area were elevated compared to mean surface water concentrations measured across Sweden (*n* = 289) of 110 ng L^–1^ ∑_24_PFAS (median 3.9 ng L^–1^).^37^ Furthermore, water concentrations in both the stream and the lake greatly exceeded the annual average (AA)-EQS of the EU Water Framework Directive of 0.65 ng L^–1^ for total PFOS, indicating that both sites were PFAS contaminated (maximum ∑PFOS 1040 ng L^–1^ at the stream (W4) and 230 ng L^–1^ at the lake (W1)).

#### Sediment

Concentrations of ∑_24_PFAS measured in sediment were 220 ng g^–1^ dw in the stream, 280 ng g^–1^ dw in the lake, and 2.4 ng g^–1^ dw in the reference pond. PFAS profiles were similar to those of water, generally with high contribution of PFOS to the total PFAS levels in the stream and the lake sediments (160 and 220 ng g^–1^ dw, respectively). Sediment concentrations were comparable to concentrations reported from a lake in Northern Sweden, which was impacted by PFAS.^38^

#### Aquatic Insects

In the lake, mean concentrations for the different groups in the lake were 400 ± 66 ng g^–1^ dw (*n* = 5) for dragonfly larvae, 720 ng g^–1^ dw (*n* = 1) for damselfly larvae, 750 ng g^–1^ dw (*n* = 1) for midges, and 660 ng g^–1^ dw (*n* = 1) for caddisfly larvae. Aquatic insect larvae that were sampled in fall 2017 had high concentration of ∑_24_PFAS in the stream and lake, with means of 2000 ± 610 ng g^–1^ dw (*n* = 4) and 520 ± 160 ng g^–1^ dw (*n* = 8), respectively, and the reference pond had 12 ± 7.1 ng g^–1^ dw (*n* = 4) (Tables S7 and S8 in Supporting Information). Differences among sites in internal PFAS concentrations correspond to differences in water and sediment concentrations. In general, PFAS congener profiles showed that short- and medium-chain PFAS were more prominent in water, whereas PFOS and other long-chain PFAS were more prominent in biota (see Figures S3–5 in Supporting Information). Dragonfly concentrations were higher in this study than previously reported for dragonflies caught at six sites (including industrial sites) in South Africa (0.32 and 9.3 ng g^–1^ ww ∑_8_PFAS).^39^ The isotope ratios δ^13^C and δ^15^N (mean values ± 1 SD) from pooled samples of each invertebrate group per sampling site (see Methods) were, for δ^13^C, −31 ± 1.2 ‰ for all sample groups (*n* = 7) at the stream and −30 ± 1.2 ‰ at the lake (*n* = 10), and for δ^15^N 17 ± 2.1 ‰ at the stream (*n* = 7) and 13 ± 1.3 ‰ at the lake (*n* = 10) (Figures S7, S8, and S9 in Supporting Information).

#### Emergent Aquatic Insects

Emergent aquatic insects showed lower, but still relatively high, PFAS concentrations than aquatic insect larvae. At the stream, a mean of 700 ng g^–1^ dw ∑_24_PFAS was determined for all samples from deposition traps, where midges made up all the captured insects. Dragonflies and damselflies caught by sweep net at the stream had concentrations of 230 ± 4.8 ng g^–1^ dw (*n* = 2) and 1200 ng g^–1^ dw ∑_24_PFAS (*n* = 1), respectively (Tables S7 and S8). At the lake, mean concentration for some insect groups from 2017 and 2018 were 160 ± 180 ng g^–1^ dw in midges (*n* = 11), 1040 ± 480 ng g^–1^ dw in alderflies (*n* = 5), and 170 ± 60 ng g^–1^ dw ∑_24_PFAS (*n* = 5) in damselflies. Mean concentrations of ∑_24_PFAS in emergent aquatic insects from deposition traps were 820 ± 590 ng g^–1^ dw (*n* = 7) in 2017 and 230 ± 260 ng g^–1^ dw in 2018 (*n* = 16). The sample with the highest PFAS concentration, 1900 ng g^–1^ dw, was alderflies from the lake in 2017 (D24 in Table S8 in Supporting Information). Compared to PFAS concentrations in aquatic larvae, concentrations in adult emerged insects were lower, e.g., four times lower in damselflies. Dragonflies that were caught in sweep nets had mean ∑_24_PFAS concentration of 130 ± 48 ng g^–1^ dw (*n* = 2) at the lake. The mean concentration of adult dragonflies from lake Söderhavet was similar to concentrations found in adult dragonflies at a pond in southern Sweden where AFFF had contaminated the water (∑_24_PFAS 270 ± 190 ng g^–1^ dw).^33^ However, in the current study, damselfly concentrations were lower than at the AFFF affected site which had average ∑_24_PFAS of 1600 ± 1300 ng g^–1^ dw.^33^ Body burden in individuals from the lake, determined by multiplying the mean ∑_24_PFAS concentration with the dry weight of the pooled sample and dividing by the number of individuals of each pooled sample, was on average 5 ng for adult damselfies and 16 ng for adult dragonflies in this study. Emergent insects from the reference pond showed a mean concentration of 10 ± 4.7 ng g^–1^ dw (*n* = 9). The mean δ^13^C in emergent aquatic insects was around 30 ‰ (−29 ± 1.2 ‰ K1, *n* = 3; −30 ± 1.9 ‰ KS, *n* = 8, Figures S7 and S8 in Supporting Information), similar to those of aquatic larvae.

#### Terrestrial Consumers

**Figure 3:**
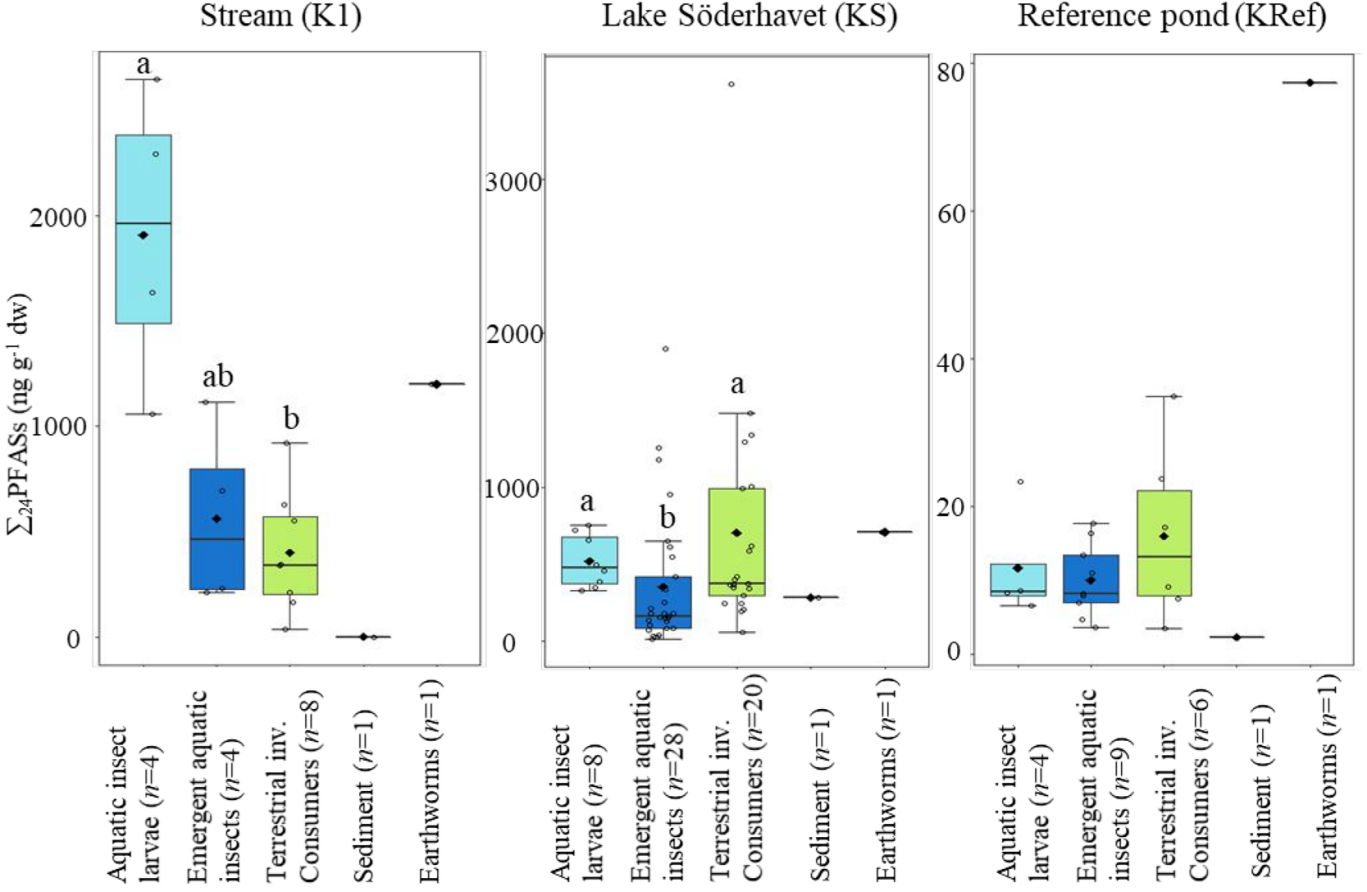
Sum of 24 PFAS (ng g^–1^ dw) in different groups from the stream (K1), the lake (KS), and the reference pond (KRef). The groups aquatic insect larvae, sediment, and earthworms were collected in fall 2017; terrestrial invertebrate consumers in spring 2017; and emergent aquatic insect in spring 2017 and 2018. Circles represent data points, diamonds represent mean values, and the band represents the median. Lower and upper hinges correspond to the first and third quantiles (25th and 75th, respectively). The lower and upper whiskers extend from the hinge to the smallest and the largest value no further than 1.5 times the interquartile range (IQR). Groups at each site that do not share the same letters have significantly different concentrations (Dunn’s test, *p* < 0.05, no significant differences were found in Kref). Sediment and earthworms were excluded from the statistical analysis due to too few samples. Note the differences in scales between KRef and the other two sites.

Mean ∑_24_PFAS concentrations (ng g^–1^ dw ±1 SD) of all terrestrial consumers were 404 ± 270 (*n* = 8) at the stream, 708 ± 770 (*n* = 21) at the lake, and 16 ± 11 (*n* = 6) at the reference pond (Figure 3, Table 1). PFAS levels in terrestrial consumers differed substantially among invertebrate groups, as indicated by high SDs. Factors that explain this variation are likely differences in exposure levels due to consumer trophic position (δ^15^N) and proportion of aquatic diet of predators (δ^13^C). Spiders, a typical invertebrate top-consumer, had highest values for δ^15^N and highest ∑_24_PFAS concentration with 630 ng g^–1^ dw at the stream and a mean of 1400 ± 82 ng g^–1^ dw at the lake site (Tables 1 and S7 and S8 in Supporting Information) among the collected terrestrial invertebrates in this study. When comparing isotope ratios of terrestrial consumers to aquatic insect larvae and emergent aquatic insects, δ^13^C was on average higher (−26 ± 0.3 ‰ K1, *n* = 8; −27 ± 1.3 ‰ KS, *n* = 19) and δ^15^N was on average lower (9 ± 3.5 ‰ K1, *n* = 8; 5 ± 2.9 ‰ KS, *n* = 19), indicating a clear difference between the aquatic and terrestrial ecosystem.

**Table 1:** Calculated Emergent Aquatic Insect Numbers, Emergence Biomass, and Insect-Mediated PFAS Transfer Per Square Meter and Day, as well as PFAS Concentration Measured in Terrestrial Spiders, for 2017 and 2018 from the Stream (K1), the Lake (KS), and the Reference Pond (KRef)^a^

	number of individuals (m^–2^ d^–1^)		biomass (mg dw m^–2^ d^–1^)		PFAS transfer (ng m^–2^ d^–1^)		PFAS in spiders (ng g^–1^ dw)
site	2017	2018	2017	2018	2017	2018	2017
K1	-	11	-	3.1	-	2.1	640
KS	25	17	49	6.4	280	23	1400 ± 82
KRef	46	18	44	9.2	0.4	0.1	35

a Due to low emergence, no values could be determined for K1 in 2017. Note that the data on emergent aquatic insects are based on insects caught via deposition traps only.

#### Among-Site Comparison of Different Biota Groups

At the stream, ∑_24_PFAS in aquatic insect larvae were somewhat higher than in emergent aquatic insects and significantly higher to terrestrial consumer (Dunn’s test *p* = 0.02) (Figure 3). At the lake, the ∑_24_PFAS concentrations of emergent aquatic insects were significantly lower than aquatic insect larvae and terrestrial consumers (*p* = 0.003) (Figure 3). PFAS profiles in biota showed enrichment of PFOS compared to other PFAS (74–93% of the total concentrations of PFAS), and PFO were especially enriched in samples collected at the lake (>87%) (Figures S3–5 in Supporting Information). Furthermore, branched PFOS isomers were enriched in the surface water (30–35% to the total PFOS concentrations), whereas lower proportions were found in all biota (0–20%) indicating faster elimination rate of branched isomers in biota, as mentioned in previous studies (Figure S6 in Supporting Information).^31,32,40^

#### Stable Isotopes between the Aquatic and Terrestrial System

On average, δ^13^C and δ^15^N were significantly different between aquatic insect larvae and terrestrial invertebrate consumers at both the stream and the lake site (Dunn post hoc *p* < 0.05; Table S11 in Supporting Information), indicating differences in diet and trophic positions.^23,41^ Aquatic insects were more depleted in δ^13^C and enriched in δ^15^N than terrestrial consumers, whereas δ^13^C of emergent aquatic insects were located in between, suggesting a mixed diet (Figures S7–S8 in Supporting Information). This can be expected since adult dragonflies, making up a large portion of the samples, prey on both emerged aquatic and terrestrial prey (Table S11 and Figures S7–S9 in Supporting Information). The more depleted δ^13^C for most terrestrial invertebrate consumers indicates predominately terrestrial food sources, but two web-building spider samples showed similar ratios as the aquatic samples, indicating high consumption of aquatic prey (Figure S8 in Supporting Information). Furthermore, ratios of δ^15^N have increasingly been used to quantify the biomagnification of contaminants, since there is a positive relationship between concentration of contaminants (e.g., POPs and MeHg) and trophic position.^10,25^ In this study, near-significant positive relationships between δ^15^N and ∑PFOS for emergent aquatic insects (*R*^2^ = 0.559, *n* = 6, *p* = 0.09) and terrestrial consumers (*R*^2^ = 0.208, *n* = 13, *p* = 0.12) from the lake (Figure S11 in Supporting Information) indicate a higher nitrogen ratio in terrestrial consumers feeding on aquatic prey, and/or higher PFAS uptake at higher trophic levels, as found in our previous study.^33^

### Insect Emergence and Transfer of PFAS from Water to Land

#### Emergence at Each Site

At lake Söderhavet, the mean number of deposited emergent insects was 25 individuals per m^–2^ d^–1^ in 2017 and 17 per m^–2^ d^–1^ in 2018 (Table 1). There was a large difference between years in mean emergence biomass deposited around the lake, with 49 mg dw m^–2^ d^–1^ in 2017 and 6.4 mg dw m^–2^ d^–1^ in 2018. Based on the study period (63 days), annual biomass was estimated to be 3.2 and 0.4 g dw m^–2^ y^–1^ in 2017 and 2018, respectively, which was within the range of what was found previously in Finland.^7,42^ At the stream site (K1), emergence was generally low in spring 2017, and biomass captured was therefore not sufficient for chemical analysis. In 2018, there was sufficient biomass for PFAS analysis of one pooled sample of midges (11 insects per m^–2^ d^–1^, Table 1). Based on this, the deposition of emergent aquatic biomass at the stream site was estimated to be 3.1 mg dw m^–2^ d^–1^, which was lower compared to that in the lake (6.4 mg dw m^–2^ d^–1^) and reference pond (9.2 mg dw m^–2^ d^–1^) for 2018. At the reference pond, aquatic insect deposition was similar to that of the lake 44 mg dw m^–2^ d^–1^ in 2017 and 9.2 mg dw m^–2^ d^–1^ in 2018, which indicates similar productivity.

#### PFAS Transfer

PFAS transfer from the lake via emergent aquatic insects was estimated to be 280 and 23 ng ∑_24_PFAS m^–2^ d^–1^ in 2017 and 2018, respectively. The total amount of PFAS deposited on the whole lake riparian zone (3 m in width) was estimated to be 101 mg ∑_24_PFAS for the sampling period (May 31 to June 15) in 2017 and 8 mg ∑_24_PFAS from May 2 to July 3 in 2018, showing substantial seasonal (during early summer peak emergence) transfer to the riparian zone, but also high between-year variation in this transfer. Little or no emergence of aquatic insects can be expected for the rest of the year (October–April).

**Figure 4:**
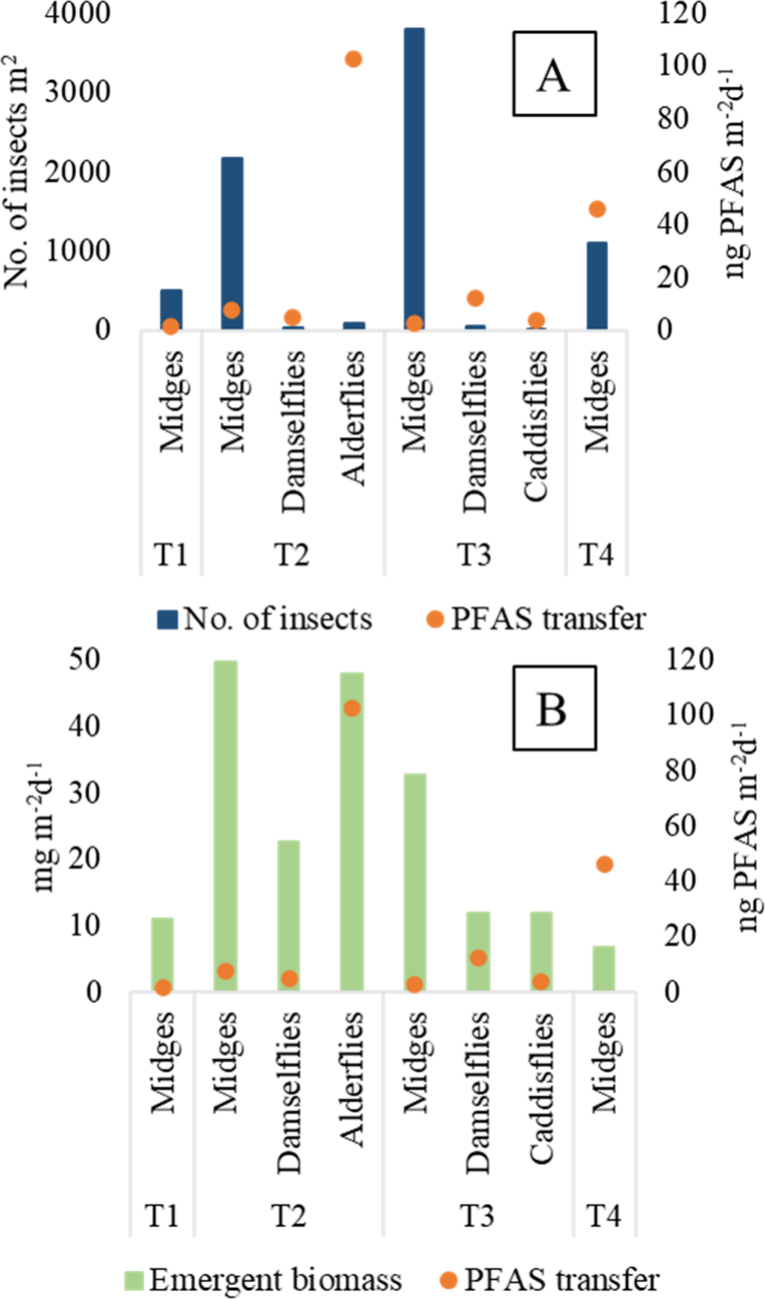
(A) Total number of emergent insects per m^2^ collected during each time period T1–T4 (blue bars) and (B) total emergent biomass mg per m^2^ during time periods T1–T4 (green bars) along with the estimated amount of daily transfer of PFAS (ng ∑_24_PFAS per m^2^) for each insect group at the riparian zone of Lake Söderhavet 2018. Only samples collected from deposition traps were used.

In 2018, traps were collected during four occasions (T1–T4, May–July), and differences between time periods at the lake can be investigated. Here, the highest biomass and ∑_24_PFAS transfer (34 mg m^–2^ d^–1^ and 91 ng m^–2^ d^–1^, respectively) occurred during T2, whereas the highest number of individuals emerged during T3 (Table S9 in Supporting Information). In other words, mid-May to mid-June (Table S9 in Supporting Information and Figure 4). When looking at different insect taxa, alderflies emerged earlier (during T2) than damselflies and caddisflies (mainly during T3 Figure 4). Midges emerged throughout the study period, but showed a peak in emergence during T3. The highest number of individuals emerged (m^–2^ d^–1^) were midges, which was expected based on results from previous studies.^7,8^ Across insect groups, the daily biomass transfer was 3–14 times lower in 2018 than in 2017. This could be explained by spring 2018 being exceptionally warm, especially in May when our sampling occurred, as local weather conditions could play an important role for insect emergence. Since the PFAS transfer was highest during T2 when the biomass of the alderflies was substantial, alderflies could be important for biodriven PFAS transfer in this area. When comparing PFAS transfer per insect group (in ng ∑_24_PFAS m^–2^ d^–1^), the rank order was as follows: alderflies (140) > midges (113) > stoneflies (17) > damselflies (6) in 2017 and alderflies (21) > damselflies (3.8) > midges (3.5) > caddisflies (1) in 2018 (Table 2).

**Table 2:** Total Emergent Aquatic Insect Number of Each Insect Group, Biomass, and Insect-Mediated PFAS Transfer per Day and Square Meter Collected at Lake Söderhavet (KS) in 2017 and 2018^a^

	Year	Total number of individuals	Number of individuals (m^–2^ d^–1^)	Biomass (mg dw m^–2^ d^–1^)	PFAS transfer (ng m^–2^ d^–1^)
Midges	2017	358	77	96	112
Midges	2018	3653	33	6.6	3.6
Alderflies	2017	50	11	46	140
Alderflies	2018	36	1.5	9.9	21
Damselflies	2017	11	2.4	23	5.8
Damselflies	2018	32	0.6	6.5	3.8
Stoneflies	2017	3	0.6	14	17
Caddisflies	2018	5	0.1	3.0	1.0

a The sum of each group in each year corresponds approximately the total PFAS transfer. Note that data on emergent aquatic insects are based on insects caught via deposition traps only.

Transfer of ∑_24_PFAS from the stream to the riparian zone was estimated to be on average 2.1 ng m^–2^ d^–1^ and for the whole study period (63 days) 5 mg ∑_24_PFAS in 2018. The transfer estimate at the stream was lower than at the lake, despite higher concentration in the surface water (1550 ng L^–1^ for ∑_24_PFAS in spring 2018). Therefore, the low emergence from the stream limited transfer of PFAS at that site (Figure 1, scenario D). In general, in terms of aquatic insect emergence, streams are more productive than similar-sized lakes,^9^ but due to the much smaller area of the stream site in this study, the low PFAS transfer due to low emergence is not surprising. Alternatively, low aquatic insect emergence and thus lower subsequent transfer to land and uptake in riparian consumers could have been caused by PFAS concentrations in the stream surpassing toxic thresholds (Figure 1, scenario C). However, concentrations were most likely not high enough to cause reduced emergence due to mortality (48 h LC_50_ of PFOS is at high ppm levels^43^), although negative effects on aquatic insects might have occurred to some extent, as the estimated no-effects value used by Environment Canada (491 ng L^–1^ in water) for aquatic organisms was exceeded.^44^ Hence, rather than acute effects, potentially negative effects on emergence could be caused by chronic exposure of relatively low PFAS concentration over time.

At the reference pond, the PFAS transfer was substantially lower compared to the other sites, with 0.4 and 0.1 ng ∑_24_PFAS m^–2^ d^–1^ in 2017 and 2018, respectively. The fact that there was a measurable PFAS transfer at the reference site, which represents PFAS background levels in the environment, confirms that PFAS are ubiquitously detectable, possibly due to atmospheric deposition or other diffuse sources.

#### Impacts on Terrestrial Consumers

Although PFAS concentrations in surface water and aquatic insect larvae were lower in the lake than in the stream, aquatic insect biomass deposited on land, total PFAS transferred from water to land, and concentrations in terrestrial consumers were higher at the lake. This is likely an effect of terrestrial consumers at the lake having a higher proportion of aquatic diet, caused by high availability of aquatic prey (Figure 1, scenario B). Conversely, spiders at the stream would mainly feed on terrestrial prey due to low availability of emergent aquatic insects, thus averting exposure to PFAS originating from the stream (Figure 1, scenario D). At the reference pond, despite low PFAS concentration in the pond water, terrestrial consumers had measurable internal ∑_24_PFAS concentrations most likely attributable to high prey availability. Hence, as for the lake and the reference pond, PFAS uptake in the terrestrial food web was magnified by high aquatic insect emergence (Figure 1, scenario B). As such, aquatic system productivity, in terms of insect emergence, seems to strongly determine PFAS transfer into terrestrial food webs (Figure 1, scenarios A, B, and D). In general, the between-year variation in emergence and thus PFAS transfer in the study region were substantial. Hence, temporal variability in insect emergence and PFAS contamination levels may interact to influence transfer and uptake of PFAS in terrestrial systems. More studies of different scenarios (Figure 1) are therefore needed, to understand such biodriven transfer of PFAS.

### Implications

The substantial quantitative transfer of PFAS from water to land via emergent aquatic insects, i.e., 102 mg ∑_24_PFAS at the lake in 2017, can have large implications for terrestrial insectivores. Considering that emergent aquatic insects can account for 50–90% of the monthly energy budget for songbirds during the defoliation period^45^ and some insectivorous birds, e.g., tree swallows, can consume up to 2000 emergent aquatic insects per day,^46^ birds can accumulate up to hundreds of nanograms PFAS per day through their diet and potentially ingest milligrams of PFAS during a emergence period. Accordingly, PFAS concentrations as high as 270 and 10,380 ng g^–1^ ww PFOS have been reported in eggs of tree swallows around the Mississippi River in the US^47^ and of great tits close to a fluorochemical plant in Belgium, respectively.^48^

In a broader context, biodriven transfer around contaminated surface waters might be comparable to atmospheric wet deposition of PFAS, and therefore play an important role at a local and regional scale, given the large number of PFAS-contaminated sites.^49,50^ In this study, the biodriven deposition of PFOS at Lake Söderhavet during the emergence periods of 2017 (250 ng m^–2^ d^–1^) and 2018 (19 ng m^–2^ d^–1^) was within the range of rain deposition rates (10–510 ng PFOS m^–2^ d^–1^) found in 28 cities in China,^51^ and higher than rain deposition rates (0.1–12 ng m^–2^ d^–1^) found in a semirural area in Germany^52^ and PFOS wet deposition at various sites around Sweden (0.057–7.4 ng m^–2^ d^–1^)^53,54^ (Table S10). This indicates that biodriven transfer via emergent aquatic insects can be similar to or even higher than rain deposition at a local scale. Moreover, because biodriven transfer to a large part consists of prey, this PFAS transfer is likely more readily incorporated into terrestrial consumer food webs.

## Supporting Information Available

Instrumental analysis, detailed sample lists, PFAS concentration (PDF)
