# Supplementary material for: Quantification of Biodriven Transfer of Per- and Polyfluoroalkyl Substances from the Aquatic to the Terrestrial Environment via Emergent Insects
Source: Environ Sci Technol. 2021 May 24;55(12):7900–9. doi: 10.1021/acs.est.0c07129 (PMC8277127; doi:10.1021/acs.est.0c07129)
Supplement: Supplementary file 1 — es0c07129_si_001.pdf [file es0c07129_si_001.pdf]

Supporting information

Quantification of Biodriven Transfer of Per- and Polyfluoroalkyl Substances from the Aquatic to the Terrestrial Environment via Emergent Insects

Alina Koch[1], Micael Jonsson[2], Leo W.Y. Yeung[1], Anna Kärrman[1], Lutz Ahrens[3], Alf Ekblad[1], Thanh Wang[1]*

[1]Man-Technology-Environment Research Centre, Örebro University, 70182 Örebro, Sweden
[2]Dept. of Ecology and Environmental Sciences, Umeå University, 90187 Umeå, Sweden
[3]Dept. of Aquatic Sciences and Assessment, Swedish University of Agricultural Sciences (SLU), Box 7050, 75007 Uppsala, Sweden

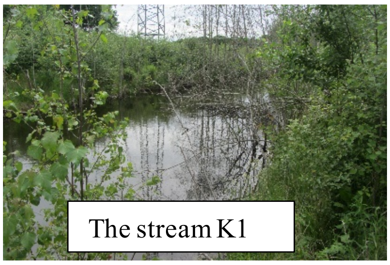

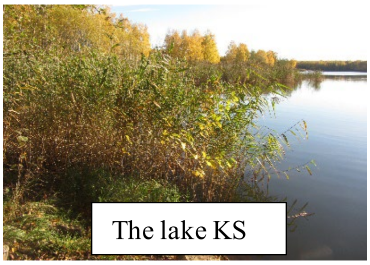

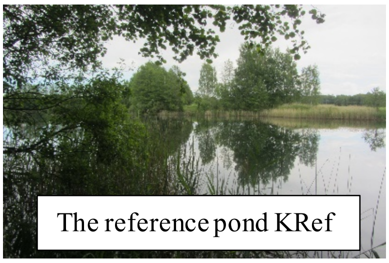

Number of pages: 31

Number of tables: 11

Number of figures: 10

Corresponding author:
Alina Koch
Doctoral student
MTM Research Centre
School of Science and Technology
Örebro University
SE-70182, Örebro
Sweden

Alina_koch@posteo.net

Table of content

Chemical analysis

Study site S1

Extraction of water samples S1

Extraction of solid samples S1

Instrumental analysis S2

Stable isotope analysis S2

Data analysis S3

Tables

Table S1. Physical and chemical water parameters S4

Table S2. List of all samples S5

Table S3. Target PFAS listed S8

Table S4. Concentrations of QC samples S9

Table S5. Method detection limits (MDL) and method quantification limits (MQL) S10

Table S6. Recovery S11

Table S7. Concentrations of all PFSA S14

Table S8. Concentrations of target PFCA, FOSA and FTSA S17

Table S9. Emergent aquatic insect number, biomass, and insect-mediated transfer S19

Table S10. Comparison of emergent insect deposition/transfer of PFOA and PFOS S20

Table S11. Results from Dunn post hoc test on differences in δ15N, δ13C and ∑24PFAS S21

Figures

Figure S1. PFAS surface water concentrations S22

Figure S2. PFAS profiles of surface water samples S23

Figure S3. PFAS profiles of pooled aquatic insect larvae samples S24

Figure S4. PFAS profiles of pooled emergent aquatic insect samples S25

Figure S5. PFAS profiles of pooled terrestrial invertebrate consumer samples S26

Figure S6. Percentage contribution of branched PFOS isomers to the total concentration of PFOS S27

Figure S7. Values (± 1 SE) of 13C vs. 15N for site K1 S28

Figure S8. Values (± 1 SE) of 13C vs. 15N for site KS S29

Figure S9. Values (± 1 SE) of 13C vs. 15N for site KRef S30

Figure S10. The sum of PFOS concentrations plotted against δ15N values of emergent aquatic insects (plot A) and terrestrial consumers (plot B) from the lake (KS) S31

References S32

Study side and chemical analysis

Study side

Since the 1940s, various industries have been active in this area, among others, recovery of shale oils and production of ammonia. Consequently, a 157 m high slagheap and several open pit-mining lakes have been constructed. The lakes and surrounding land became, to a large extent, polluted by petroleum hydrocarbon and heavy metals, but also by e.g. polycyclic aromatic hydrocarbons, solvents, PCBs, and dioxins.[1] Currently, paper and pigment industries, as well as Sweden's only hazardous waste management facility, are active in this area. From this facility, wastewater is treated on site and the effluent water together with cooling waters and runoff, about 1.5 million m^3 water per year (calculated for an average year), are released into a small stream (Ulfstorpsbäcken) that enters a pit lake named Söderhavet after 1.2 km.[2] Along the stream is a 500 m stretch of sewage sludge deposit from the municipality's wastewater treatment plant. In 2015, an investigation of fish in lake Söderhavet found high PFOS concentrations with an average of 750 ng g^{-1} wet weight (ww), providing the first report on PFAS contamination at Kvarntorp.[3] $\sum_{24}$PFAS concentrations from samples collected in 2016 were 156 ng L^{-1} in surface water and 292 ng g^{-1} dry weight (dw) in aquatic invertebrates from Söderhavet.[4]

Extraction of water samples

Surface water samples (0.5 L) were filtered through GFF filters (Whatman GF/C; diameter 47 mmm, 1.2 µm pore size, pre-baked at 450 °C for 3 h). The filters were transferred into 15-mL PP tubes and about 4 mL methanol was added followed by a 15-min sonication. The supernatant of the filter extract was then added to the respective water sample. The water samples were spiked with 1 ng internal standard (IS) mixture and extracted by solid phase extraction (SPE) with weak-anion exchange (WAX) cartridges (Waters 150 mg, 6 mL, 30 µm). The WAX cartridges were preconditioned by 4 mL 0.1% NH_4OH in methanol, 4 mL methanol and 4 mL MilliQ water. Then, the samples were loaded and passed through the cartridges with 2-3 drops per second. The sample containers were finally rinsed with 2 mL methanol and added onto the cartridges. Afterwards, the cartridges were washed (4 mL ammonium acetate buffer) and dried. PFAS were eluted by 4 mL methanol and with 4 mL of 0.1% NH_4OH in methanol collected in a PP tube.

Extraction of solid samples

All sorted and pooled invertebrates and sediment samples were freeze-dried and homogenised with a mortar and pestle or with a stainless-steel ball mill (Retsch mixer mill MM200). For sediment, 500 mg, and for invertebrates, the available biomass from the pooled samples (40-150 mg) were weighed into 15 mL PP tubes. Then 1 ng IS mixture was added, followed by alkaline digestion (2 mL of 0.2 M NaOH in

methanol) and solid-liquid extraction (2 x 4 mL methanol), based on Powley, et al. [5]. Afterwards, the extracts were concentrated to <1 mL in an automatic evaporator (RapidVap vacuum dry evaporation, Labconco Corporation, Kansas City, US) and then diluted with MilliQ water (1:9) for SPE clean-up. The SPE procedure was preconditioning and loading the samples as it was done for the water samples. The washing step prior to elution was this time with 2 x 4 mL ammonium acetate buffer and 6 mL MilliQ water to retrieve cleaner extracts. Simultaneously, Envi Carb cartridges (Sulpelco) were pre-conditioned with 3 x 1 mL methanol. Then SPE cartridges were placed on top of the Envi Carb cartridges for a tandem elution with 4 mL methanol and with 4 mL of 0.1% NH_4OH in methanol. All extracts were concentrated in the RapidVap until <0.3 mL and then transferred to LC vials. The PP tubes were rinsed with methanol, which was then added to the LC vial. The final extracts were then concentrated under nitrogen and spiked with 1 ng recovery standard.

Since some invertebrate samples were collected in traps containing glycol, the glycol was tested for potential leaching effects or contamination of PFAS from a few traps. The glycol was extracted using SPE and no PFAS were detected above the MDLs.

Instrumental analysis

Extracts were analysed by ultra-performance liquid chromatography coupled to a tandem mass spectrometry (UPLC-MS/MS Waters Xevo TQ-S, MA, USA). Negative electrospray was used and mobile phase consisted of methanol (B) and a 30:70 methanol:water mixture (A), in both 2 mmol L^{-1} ammonium acetate was added. A 100 mm C18 BEH reversed phased column (1.7 µm, 2.1 mm) was used to separate target PFAS. Gradient elution was used for target linear PFAS, while isocratic elution was used to reach better separation of branched isomers, mainly PFOS isomers. The elution procedure was as follows; start with 100% mobile phase A until 7 min, then increase B to 35% and held isocratic until 18 min, increase B to 90% from 18 to 22 min and finally equilibration until, 25 min. Other instrumental settings were: desolvation temperature 400 °C, desolvation gas flow 800 L h^{-1}, column temperature 50 °C, source temperature 150 °C, mobile phase flow rate 0.3 mL min^{-1}, and capillary voltage 3.0 kV.

Stable isotope analysis

Selected invertebrate samples were freeze-dried, homogenised, and then weighed into tin capsules (0.5 mg). They were then analysed for stable nitrogen ($\delta^{15}N$) and carbon ($\delta^{13}C$) isotopes with an elemental analyser coupled to an Isotope-Ratio mass spectrometer (IRMS, Vario Pyro Cube and Isoprime Precision, Elementar UK Ltd., Stockport). Values from the stable isotope analysis were reported in ratios of the heavy to the light isotope of each element, as $\delta^{13}C$ and $\delta^{15}N$ in parts per thousand (‰) and related to Vienna Pee Dee Belemnite (V-PDB) for ^{13}C and atmospheric N_2 for ^{15}N. A working standard, an in-

house wheat sample, was calibrated with IAEA-CH6 for ^{13}C and IAEA-N1 for ^{15}N and had the following ratios; -26.231‰ $\delta^{13}C$ and 5.817‰ $\delta^{15}N$. A nine-point calibration curve of the working standard was used. The precision of standard samples was 0.019‰ for δ13C and 0.013‰ for $\delta^{15}N$ (n = 13). Due to potential contamination from carbon contained in the propylene glycol (the preservative used in the pitfall traps), carbon isotope data was used for δ13C vs. $\delta^{15}N$ to show general food web structures (Figures S7-9), but was not used for subsequent investigation of terrestrial consumer diet. Important to note, this accounts only for vertebrates collected in pitfall traps, and emergent insects used for isotope analysis were only those caught by the sweep net method and not in deposition traps which contained glycol. Unfortunately, the effects of glycol on the isotope analysis have to our knowledge not yet been investigated. Therefore, in later discussions we focus mainly on spiders, since they are known to feed substantially on emergent aquatic insects.[6]

Data analysis

For quantification and recovery correction, isotopically labelled standards were used (Table S3), and three calibration curves were prepared; one for the quantification of 26 linear PFAS (seven-point calibration), one for quantification of branched PFOS isomers (five point calibration), and the third one for quantification of branched PFHxS isomers (six point calibration). Most IS recoveries were satisfactory (50-120%), although some compounds (PFBA, FOSA, 6:2 FTSA and 8:2 FTSA) were affected by either matrix suppression or enhancement in individual samples (Table S6 in SI). Furthermore, the recoveries of isotopically labelled $^{13}C_2$-FTSAs were most likely affected by interferences from natural occurring isotopes for samples with relatively high concentrations (e.g. water samples from K1). Natural occurring ^{34}S and ^{13}C isotopes contribute to M+2 isotopic masses that have relative abundance of 5-6% of the monoisotopic mass for FTSAs. These additional M+2 isotopes could increase the response for the respective internal standards, resulting in systematic bias; similar to what has been reported for cortisol.[7] Therefore quantification of FTSAs in all samples was corrected for the naturally occurring isotopes (i.e. 5.3% for 4:2, 5.4% for 6:2, 5.6% for 8:2 and 5.9% for 10:2 FTSA) and the recovery was corrected for the labelled internal standards 6:2 and 8:2 FTSA.

Tables

Table S1. Physical and chemical water parameters measured in fall 2017 in the stream (K1), at two location (K1 and K2) in the lake Söderhavet and the reference pond (KRef). TOC = total organic carbon, DOC = dissolved organic carbon.

		K1	K2	K3	KRef
Temperature	°C	15	11	11	12
pH		7.3	7.5	7.5	7.7
Conductivity	mS m25^{-1}	147	121	120	61
Alkalinity/acidity	mekv l^{-1}	1.9	2.4	2.4	3.9
Absorbance	420 nm	0.038	0.028	0.028	0.022
Absorbance	436 nm	0.029	0.021	0.021	0.017
TOC	mg l^{-1}	10.9	9.7	8.4	4.9
DOC	mg l^{-1}	12	8	9	5
Total N	µg l^{-1}	1960	1960	1900	213
NH_4	µg l^{-1}	134	160	207	11
NO_2+NO_3	µg l^{-1}	1010	1510	1460	<3
PO_4	µg l^{-1}	2	<1	<1	5
Total P	µg l^{-1}	33.4	13.4	12.5	24.5
SO_4	mekv l^{-1}	2.1	6.7	6	1.7
Cl	mekv l^{-1}	9.6	3.4	3.4	0.79
F	mg l^{-1}	1.2	0.45	0.5	0.62
Ca	mekv l^{-1}	4.9	7.5	7.5	4.7
Mg	mekv l^{-1}	0.35	1.2	1.2	0.78
Na	mekv l^{-1}	7.4	2.7	2.7	1
K	mekv l^{-1}	0.97	0.69	0.72	0.16
Si	mg l^{-1}	1.3	0.55	0.45	4

Table S2. Sample IDs, sampling sites, environmental compartment groups, sample group/invertebrate taxa (family/suborder), collected wet weight (g ww) and dry weight (g dw), number of individuals (Quantity) and the year collected.

ID	Sampling site	Group	Group/Taxa	g ww	g dw	Quantity	Year
W1	K1	Water	Water 1_S17				2017
W2	KS	Water	Water S_S17				2017
W3	KRef	Water	Water Ref_S17				2017
W4	K1	Water	Water 1_F17				2017
W5	KS	Water	Water S_F17				2017
W6	KRef	Water	Water Ref_F17				2017
W7	K1	Water	Water 1_S18				2018
W8	KS	Water	Water S_S18				2018
W9	KRef	Water	Water Ref_S18				2018
S1	K1	Sediment	Sed K1	250.23	154.91		2017
S2	KS	Sediment	Sed KS	226.27	166.15		2017
S3	KRef	Sediment	Sed Ref	265.08	180.86		2017
L1	KS	Aquatic insect larvae	*Diptera Nematocera*	0.12	0.03	59	2017
L2	KS	Aquatic insect larvae	*Trichoptera*	0.24	0.06	58	2017
L3	KS	Aquatic insect larvae	*Odonata Zygoptera*	0.19	0.05	30	2017
L4	KS	Aquatic insect larvae	*Odonata Anisoptera*	0.26	0.06	9	2017
L5	KS	Aquatic insect larvae	*Odonata Anisoptera*	0.65	0.13	2	2017
L6	KS	Aquatic insect larvae	*Odonata Anisoptera*	0.63	0.14	3	2017
L7	KS	Aquatic insect larvae	*Odonata Anisoptera*	0.34	0.07	2	2017
L8	KS	Aquatic insect larvae	*Odonata Anisoptera*	0.68	0.14	6	2017
L9	K1	Aquatic insect larvae	*Odonata Zygoptera*	0.30	0.08	69	2017
L10	K1	Aquatic insect larvae	*Odonata Anisoptera*	0.18	0.05	8	2017
L11	K1	Aquatic insect larvae	*Odonata Anisoptera*	1.76	0.25	3	2017
L12	K1	Aquatic insect larvae	*Odonata Anisoptera*	0.98	0.36	4	2017
L13	KRef	Aquatic insect larvae	*Diptera Nematocera*	0.61	0.11	165	2017
L14	KRef	Aquatic insect larvae	*Megaloptera Sialidae*	0.15	0.03	8	2017
L15	KRef	Aquatic insect larvae	*Odonata Zygoptera*	0.38	0.08	19	2017
L16	KRef	Aquatic insect larvae	*Odonata Anisoptera*	1.08	0.20	6	2017
E1	K1	Earthworms	*Megadrilacea*	12.98	2.84	~50	2017
E2	KS	Earthworms	*Megadrilacea*	7.88	1.24	~30	2017
E4	KRef	Earthworms	*Megadrilacea*	8.25	1.37	~20	2017
SW1	K1	Emergent aquatic insects	*Odonata Zygoptera*	0.56	0.17	26	2018
SW2	K1	Emergent aquatic insects	*Odonata Anisoptera*	1.07	0.38	3	2018
SW3	K1	Emergent aquatic insects	*Odonata Anisoptera*	0.58	0.17	2	2018
SW4	KS	Emergent aquatic insects	*Diptera Nematocera*	0.23	0.18	365	2018
SW5	KS	Emergent aquatic insects	*Diptera Nematocera*	0.49	0.28	658	2018
SW6	KS	Emergent aquatic insects	*Diptera Nematocera*	0.33	0.20	524	2018
SW7	KS	Emergent aquatic insects	*Odonata Anisoptera*	1.32	0.44	4	2018
SW8	KS	Emergent aquatic insects	*Odonata Anisoptera*	1.23	0.44	3	2018

SW9	KS	Emergent aquatic insects	*Odonata Zygoptera*	0.34	0.14	13	2018
SW10	KRef	Emergent aquatic insects	*Diptera Nematocera*	0.48	0.29	426	2018
SW11	KRef	Emergent aquatic insects	*Odonata Zygoptera*	0.26	0.11	10	2018
SW12	KRef	Emergent aquatic insects	*Megaloptera Sialidae*	0.11	0.04	7	2018
SW13	KRef	Emergent aquatic insects	*Odonata Anisoptera*	0.72	0.25	2	2018
SW14	KRef	Emergent aquatic insects	*Odonata Anisoptera*	0.74	0.27	3	2018
D1	K1	Emergent aquatic insects	*Diptera Nematocera*	0.71	0.18	617	2018
D2	KS	Emergent aquatic insects	*Diptera Nematocera*	0.11	0.04	147	2018
D3	KS	Emergent aquatic insects	*Diptera Nematocera*	0.27	0.06	327	2018
D4	KS	Emergent aquatic insects	*Diptera Nematocera*	0.17	0.05	230	2018
D5	KS	Emergent aquatic insects	*Diptera Nematocera*	0.50	0.14	284	2018
D6	KS	Emergent aquatic insects	*Diptera Nematocera*	0.30	0.09	787	2018
D7	KS	Emergent aquatic insects	*Diptera Nematocera*	0.25	0.07	697	2018
D8	KS	Emergent aquatic insects	*Diptera Nematocera*	0.31	0.08	612	2018
D9	KS	Emergent aquatic insects	*Diptera Nematocera*	0.90	0.20	569	2018
D10	KRef	Emergent aquatic insects	*Diptera Nematocera*	0.59	0.14	388	2018
D11	KRef	Emergent aquatic insects	*Diptera Nematocera*	2.22	0.49	1002	2018
D12	KS	Emergent aquatic insects	*Trichoptera*	0.33	0.10	5	2018
D13	KRef	Emergent aquatic insects	*Megaloptera Sialidae*	0.27	0.07	10	2018
D14	KS	Emergent aquatic insects	*Megaloptera Sialidae*	0.39	0.10	14	2018
D15	KS	Emergent aquatic insects	*Megaloptera Sialidae*	0.37	0.09	14	2018
D16	KS	Emergent aquatic insects	*Megaloptera Sialidae*	0.17	0.05	8	2018
D17	KS	Emergent aquatic insects	*Odonata Zygoptera*	0.42	0.11	12	2018
D18	KS	Emergent aquatic insects	*Odonata Zygoptera*	0.26	0.07	6	2018
D19	KS	Emergent aquatic insects	*Odonata Zygoptera*	0.29	0.09	7	2018
D20	KS	Emergent aquatic insects	*Odonata Zygoptera*	0.20	0.06	7	2018
D21	KS	Emergent aquatic insects	*Diptera Nematocera*	0.40	0.10	129	2017
D22	KS	Emergent aquatic insects	*Diptera Nematocera*	0.57	0.11	149	2017
D23	KS	Emergent aquatic insects	*Diptera Nematocera*	0.49	0.24	80	2017
D24	KS	Emergent aquatic insects	*Megaloptera Sialidae*	0.54	0.10	23	2017
D25	KS	Emergent aquatic insects	*Megaloptera Sialidae*	0.59	0.11	27	2017
D26	KS	Emergent aquatic insects	*Odonata Zygoptera*	0.41	0.11	11	2017
D27	KS	Emergent aquatic insects	*Plecoptera*	0.28	0.06	3	2017
D28	KRef	Emergent aquatic insects	*Diptera Nematocera*	0.79	0.21	214	2017
P1	K1	Terrestrial invertebrate consumers	*Araneae*	1.59	1.26	94	2017
P2	K1	Terrestrial invertebrate consumers	*Araneae*	0.38	0.14	13	2017
P3	K1	Terrestrial invertebrate consumers	*Coleoptera*	8.41	3.07	36	2017
P4	K1	Terrestrial invertebrate consumers	*Coleoptera*	4.01	1.46	69	2017
P5	K1	Terrestrial invertebrate consumers	*Coleoptera*	0.27	0.91	23	2017
P6	K1	Terrestrial invertebrate consumers	*Hymenoptera Formicidae*	0.37	0.12	133	2017
P7	K1	Terrestrial invertebrate consumers	*Julida*	3.17	1.38	27	2017
P8	K1	Terrestrial invertebrate consumers	*Lithobiomorpha*	1.07	0.35	92	2017
P9	KS	Terrestrial invertebrate consumers	*Araneae*	0.48	0.15	190	2017

P10	KS	Terrestrial invertebrate consumers	*Araneae*	0.92	0.32	107	2017
P11	KS	Terrestrial invertebrate consumers	*Araneae*	1.16	0.34	182	2017
P12	KS	Terrestrial invertebrate consumers	*Coleoptera*	5.51	2.23	12	2017
P13	KS	Terrestrial invertebrate consumers	*Coleoptera*	6.29	2.40	14	2017
P14	KS	Terrestrial invertebrate consumers	*Coleoptera*	8.84	3.53	23	2017
P15	KS	Terrestrial invertebrate consumers	*Coleoptera*	0.66	0.27	16	2017
P16	KS	Terrestrial invertebrate consumers	*Coleoptera*	0.73	0.21	21	2017
P17	KS	Terrestrial invertebrate consumers	*Coleoptera*	2.02	0.77	35	2017
P18	KS	Terrestrial invertebrate consumers	*Coleoptera*	0.58	0.23	208	2017
P19	KS	Terrestrial invertebrate consumers	*Coleoptera*	0.21	0.66	55	2017
P20	KS	Terrestrial invertebrate consumers	*Coleoptera*	0.46	0.16	113	2017
P21	KS	Terrestrial invertebrate consumers	*Coleoptera*	0.53	0.18	2	2017
P22	KS	Terrestrial invertebrate consumers	*Hymenoptera Formicidae*	0.17	0.07	55	2017
P23	KS	Terrestrial invertebrate consumers	*Hymenoptera Formicidae*	0.22	0.08	94	2017
P24	KS	Terrestrial invertebrate consumers	*Hymenoptera Formicidae*	0.32	0.11	160	2017
P25	KS	Terrestrial invertebrate consumers	*Julida*	1.26	0.51	30	2017
P26	KS	Terrestrial invertebrate consumers	*Julida*	2.56	1.04	40	2017
P27	KS	Terrestrial invertebrate consumers	*Julida*	1.83	0.83	41	2017
P29	KS	Terrestrial invertebrate consumers	*Lithobiomorpha*	0.20	0.07	20	2017
P30	KS	Terrestrial invertebrate consumers	*Lithobiomorpha*	0.57	0.19	61	2017
P31	KRef	Terrestrial invertebrate consumers	*Araneae*	1.31	0.36	94	2017
P32	KRef	Terrestrial invertebrate consumers	*Coleoptera*	8.69	3.51	17	2017
P33	KRef	Terrestrial invertebrate consumers	*Coleoptera*	2.36	0.81	77	2017
P34	KRef	Terrestrial invertebrate consumers	*Hymenoptera Formicidae*	0.12	0.03	40	2017
P35	KRef	Terrestrial invertebrate consumers	*Julida*	1.84	0.81	24	2017
P36	KRef	Terrestrial invertebrate consumers	*Lithobiomorpha*	0.25	0.08	6	2017

Table S3. Target PFAS listed in compound groups (PFCA, PFSA, FOSA, and FTSA), their chemical formula, parent ion, chosen quantification ion, qualification ion and the corresponding IS compound chosen for quantification.

Acronym	Compound	Chemical formula	Parent ion (m/z)	Product ion quantification (m/z)	Product ion qualification (m/z)	Corresponding IS
**PFCAs**						
PFBA	Perfluorobutanoate	$C_3F_7CO_2^-$	213	169	-	$^{13}C_4$ PFBA
PFPeA	Perfluoropentanoate	$C_4F_9CO_2^-$	263	219	-	$^{13}C_2$ PFHxA
PFHxA	Perfluorohexanoate	$C_5F_{11}CO_2^-$	313	269	119	$^{13}C_2$ PFHxA
PFHpA	Perfluoroheptanoate	$C_6F_{13}CO_2^-$	363	169	319	$^{13}C_2$ PFHxA
PFOA	Perfluorooctanoate	$C_7F_{15}CO_2^-$	413	369	169	$^{13}C_4$ PFOA
PFNA	Perfluorononanoate	$C_8F_{17}CO_2^-$	463	419	219	$^{13}C_5$ PFNA
PFDA	Perfluorodecanoate	$C_9F_{19}CO_2^-$	513	469	219	$^{13}C_2$ PFDA
PFUnDA	Perfluoroundecanoate	$C_{10}F_{21}CO_2^-$	563	269	519	$^{13}C_2$ PFUnDA
PFDoDA	Perfluorododecanoate	$C_{11}F_{23}CO_2^-$	613	169	569	$^{13}C_2$ PFDoDA
PFTrDA	Perfluorotridecanoate	$C_{12}F_{25}CO_2^-$	663	619	169	$^{13}C_2$ PFDoDA
PFTeDA	Perfluorotetradecanoate	$C_{13}F_{27}CO_2^-$	713	669	169	$^{13}C_2$ PFTeDA
PFHxDA	Perfluorohexadecanoate	$C_{15}F_{31}CO_2^-$	813	769	169	$^{13}C_2$ PFHxDA
PFOcDA	Perfluorooctadecanoate	$C_{17}F_{35}CO_2^-$	913	869	169	$^{13}C_2$ PFHxDA
**PFSAs**						
PFBS	Perfluorobutane sulfonate	$C_4F_9SO_3^-$	299	80	99	$^{18}O_2$ PFHxS
PFPeS	Perfluoropentane sulfonate	$C_5F_{11}SO_3^-$	349	80	99	$^{18}O_2$ PFHxS
PFHxS	Perfluorohexane sulfonate	$C_6F_{13}SO_3^-$	399	99	80/119	$^{18}O_2$ PFHxS
1*m*-PFHxS	1*m*- perfluorohexane sulfonate	$C_6F_{13}SO_3^-$	399	99	119	$^{18}O_2$ PFHxS
4/2*m*-PFHxS	4/2*m*- perfluorohexane sulfonate	$C_6F_{13}SO_3^-$	399	119	99	$^{18}O_2$ PFHxS
3*m*-PFHxS	3*m*- perfluorohexane sulfonate	$C_6F_{13}SO_3^-$	399	80	99	$^{18}O_2$ PFHxS
PFHpS	Perfluoroheptane sulfonate	$C_7F_{15}SO_3^-$	449	99	80	$^{13}C_4$ PFOS
PFOS	Perfluorooctane sulfonate	$C_8F_{17}SO_3^-$	499	99	80/169	$^{13}C_4$ PFOS
1*m*-PFOS	1*m*- perfluorooctane sulfonate	$C_8F_{17}SO_3^-$	499	99	169	$^{13}C_4$ PFOS
6/2*m*-PFOS	6/2*m*- perfluorooctane sulfonate	$C_8F_{17}SO_3^-$	499	169	80	$^{13}C_4$ PFOS
3/4/5*m*-PFOS	3/4/5*m*- perfluorooctane sulfonate	$C_8F_{17}SO_3^-$	499	80	99	$^{13}C_4$ PFOS
PFNS	Perfluorononane sulfonate	$C_9F_{19}SO_3^-$	549	80	99	$^{13}C_4$ PFOS
PFDS	Perfluorodecane sulfonate	$C_{10}F_{21}SO_3^-$	599	99	80	$^{13}C_4$ PFOS
PFDoDS	Perfluorododecane sulfonate	$C_{12}F_{25}SO_3^-$	699	99	80	$^{13}C_4$ PFOS
**Sulfonamide**						
PFOSA	Perfluorooctane sulfonamide	$C_8H_2F_{17}NO_2S$	499	80	169	$^{13}C_8$ PFOSA
**FTSAs**						
4:2 FTSA	4:2 fluorotelomer sulfonate	$C_6H_4F_9SO_3^-$	327	307	81	$^{13}C_2$ 6:2 FTSA
6:2 FTSA	6:2 fluorotelomer sulfonate	$C_8H_4F_{13}SO_3^-$	427	407	81	$^{13}C_2$ 6:2 FTSA
8:2 FTSA	8:2 fluorotelomer sulfonate	$C_{10}H_4F_{17}SO_3^-$	527	507	81	$^{13}C_2$ 8:2 FTSA
10:2 FTSA	10:2 fluorotelomer sulfonate	$C_{12}H_4F_{21}SO_3^-$	627	607	81	$^{13}C_2$ 8:2 FTSA

Table S4. Concentrations of QC samples (ng g^{-1}) from biota batches containing aquatic bloodworms (Chironomidae tetans). Note that the QC samples were not spiked with surrogate standard and branched isomers of PFHxS and PFOS were all below MDL.

ID	B1 QC	B2 QC	B3 QC	B4 QC	B5 QC
PFBS	6.3	7.3	4.1	5.3	5.6
PFPeS	5.7	6.4	6.8	6.5	6.2
L-PFHxS	5.7	6.1	5.9	6.2	5.9
PFHpS	5.4	6.0	6.3	6.5	6.5
L-PFOS	6.4	6.4	6.8	6.5	6.3
PFNS	5.4	6.3	5.9	5.3	4.9
PFDS	3.0	5.1	5.0	3.8	2.8
PFDoDS	1.7	4.0	4.4	2.5	0.8
PFHxA	25.2	18.7	16.4	20.8	19.6
PFHpA	6.7	6.5	6.6	7.2	6.1
PFOA	7.0	6.9	7.2	7.1	7.3
PFNA	6.8	7.0	6.7	6.8	6.8
PFDA	6.4	7.1	6.5	7.0	6.5
PFUnDA	6.2	6.7	6.8	6.9	6.6
PFDoDA	6.1	6.5	6.5	6.5	6.4
PFTrDA	4.1	5.4	5.0	4.4	3.7
FOSA	7.9	7.3	6.9	7.4	7.0
6:2_FTSA	7.0	7.5	7.4	7.7	7.4
8:2 FTSA	8.0	8.5	8.6	8.6	8.2
10:2 FTSA	5.6	10.5	11.2	8.1	6.1

Table S5. Method detection limits (MDL) and method quantification limits (MQL) of the target PFAS, branched PFOS and branched PFHxS analysis for all batches analysed (ng L^{-1} for water and ng g^{-1} dw for all other). The lowest point of calibration was used when no peak was detected in the solvent blanks (P. calib.).

	Water batch- W1-9			Batch 1- S2,S3, E1-3, SW1-14			Batch 2- S1,D1-16			Batch 3- D17-20, -L1-13			Batch 4-L14-16,P1-14			Batch 5- P15-31			Batch 6- P32-36,D21-28		
	P. calib.	MDL	MQL	P. calib.	MDL	MQL	P. calib.	MDL	MQL	P. calib.	MDL	MQL	P. calib.	MDL	MQL	P. calib.	MDL	MQL	P. calib.	MDL	MQL
PFBS	0.08	0.02	0.08	0.13	0.03	0.09	0.13	0.03	0.09	0.13	0.02	0.07	0.13	0.03	0.09	0.13	0.03	0.10	0.13	0.03	0.11
PFHxA	0.08	0.08	0.27	0.13	0.13	0.44	0.13	0.05	0.18	0.13	0.03	0.09	0.13	0.03	0.09	0.13	0.13	0.44	0.13	0.13	0.44
PFHpA	0.08	0.08	0.27	0.13	0.02	0.06	0.13	0.03	0.09	0.13	0.04	0.14	0.13	0.01	0.05	0.13	0.01	0.04	0.13	0.02	0.07
PFPeS	0.08	0.08	0.27	0.13	0.00	0.01	0.13	0.13	0.44	0.13	0.13	0.44	0.13	0.13	0.44	0.13	0.13	0.44	0.13	0.13	0.44
PFHxS	0.08	0.01	0.05	0.13	0.10	0.34	0.13	0.04	0.12	0.13	0.04	0.13	0.13	0.04	0.15	0.13	0.03	0.10	0.13	0.05	0.17
PFHpS	0.08	0.08	0.27	0.13	0.13	0.44	0.13	0.13	0.44	0.13	0.13	0.44	0.13	0.01	0.03	0.13	0.13	0.44	0.13	0.13	0.44
PFOA	0.08	0.02	0.06	0.13	0.07	0.22	0.13	0.19	0.64	0.13	0.04	0.14	0.13	0.04	0.15	0.13	0.07	0.22	0.13	0.09	0.31
PFNA	0.08	0.08	0.27	0.13	0.13	0.44	0.13	0.13	0.44	0.13	0.13	0.44	0.13	0.13	0.44	0.13	0.13	0.44	0.13	0.13	0.44
FOSA	0.08	0.00	0.01	0.13	0.13	0.44	0.13	0.13	0.44	0.13	0.01	0.05	0.13	0.13	0.44	0.13	0.13	0.44	0.13	0.13	0.44
PFOS	0.08	0.08	0.27	0.13	1.05	3.51	0.13	0.13	0.44	0.13	0.13	0.44	0.13	0.05	0.17	0.13	0.07	0.25	0.13	0.21	0.70
PFOS	0.08	0.08	0.27	0.13	1.05	3.51	0.13	0.09	0.29	0.13	0.04	0.13	0.13	0.05	0.18	0.13	0.07	0.22	0.13	0.22	0.72
PFDA	0.08	0.08	0.27	0.13	0.13	0.44	0.13	0.04	0.13	0.13	0.03	0.11	0.13	0.02	0.05	0.13	0.02	0.05	0.13	0.13	0.44
PFUnDA	0.08	0.08	0.27	0.13	0.04	0.14	0.13	0.09	0.29	0.13	0.05	0.17	0.13	0.13	0.44	0.13	0.13	0.44	0.13	0.13	0.44
PFNS	0.08	0.08	0.27	0.13	0.13	0.44	0.13	0.13	0.44	0.13	0.13	0.44	0.13	0.13	0.44	0.13	0.13	0.44	0.13	0.13	0.44
PFDS	0.08	0.08	0.27	0.13	0.13	0.44	0.13	0.13	0.44	0.13	0.13	0.44	0.13	0.13	0.44	0.13	0.13	0.44	0.13	0.13	0.44
PFDoDA	0.08	0.08	0.27	0.13	0.13	0.44	0.13	0.13	0.44	0.13	0.13	0.44	0.13	0.13	0.44	0.13	0.13	0.44	0.13	0.13	0.44
PFTrDA	0.08	0.08	0.27	0.13	0.13	0.44	0.13	0.13	0.44	0.13	0.13	0.44	0.13	0.13	0.44	0.13	0.13	0.44	0.13	0.13	0.44
PFDoDS	0.08	0.08	0.27	0.13	0.13	0.44	0.13	0.13	0.44	0.13	0.13	0.44	0.13	0.13	0.44	0.13	0.13	0.44	0.13	0.13	0.44
PFTDA	0.08	0.08	0.27	0.13	0.13	0.44	0.13	0.13	0.44	0.13	0.12	0.38	0.13	0.13	0.44	0.13	0.13	0.44	0.13	0.13	0.44
PFHxDA	0.08	0.06	0.19	0.13	0.13	0.44	0.13	0.13	0.44	0.13	0.13	0.44	0.13	0.13	0.44	0.13	0.13	0.44	0.13	0.13	0.44
PFOcDA	0.08	0.08	0.27	0.13	0.13	0.44	0.13	0.13	0.44	0.13	0.13	0.44	0.13	0.13	0.44	0.13	0.13	0.44	0.13	0.13	0.44
4:2 FTSA	0.08	0.08	0.27	0.13	0.13	0.44	0.13	0.13	0.44	0.13	0.13	0.44	0.13	0.13	0.44	0.13	0.13	0.44	0.13	0.13	0.44
6:2 FTSA	0.08	0.08	0.27	0.13	0.21	0.69	0.13	0.16	0.53	0.13	0.15	0.50	0.13	0.22	0.74	0.13	0.20	0.67	0.13	0.16	0.55
8:2 FTSA	0.08	0.08	0.27	0.13	0.13	0.44	0.13	0.13	0.44	0.13	0.13	0.44	0.13	0.13	0.44	0.13	0.13	0.44	0.13	0.13	0.44
10:2 FTSA	0.08	0.08	0.27	0.13	0.13	0.44	0.13	0.13	0.44	0.13	0.13	0.44	0.13	0.13	0.44	0.13	0.13	0.44	0.13	0.13	0.44
1m-PFOS	0.08	0.27	0.04	0.13	0.13	0.44	0.13	0.13	0.44	0.13	0.13	0.44	0.13	0.13	0.44	0.13	0.13	0.44	0.13	0.13	0.44
6/2m-PFOS	0.08	0.27	0.04	0.13	0.13	0.43	0.13	0.13	0.44	0.13	0.13	0.44	0.13	0.13	0.44	0.13	0.05	0.18	0.13	0.13	0.44
3/4/5m-PFOS	0.08	0.27	0.04	0.13	0.09	0.30	0.13	0.13	0.44	0.13	0.13	0.44	0.13	0.13	0.44	0.13	0.01	0.02	0.13	0.02	0.08
1m-PFHxS	0.08	0.08	0.27	0.13	0.13	0.44	0.13	0.13	0.44	0.13	0.13	0.44	0.13	0.13	0.44	0.13	0.13	0.44	0.13	0.13	0.44
2/4m-PFHxS	0.08	0.08	0.27	0.13	0.13	0.44	0.13	0.13	0.44	0.13	0.13	0.44	0.13	0.13	0.44	0.13	0.13	0.44	0.13	0.13	0.44
3m-PFHxS	0.08	0.08	0.27	0.13	0.13	0.44	0.13	0.13	0.44	0.13	0.13	0.44	0.13	0.13	0.44	0.13	0.13	0.44	0.13	0.13	0.44

Table S6. Recovery of each sample for PFSA (IA PFOS/PFHxS recovery of the linear isomer from isomer analysis), PFCAs, PFOSA and FTSAs.

ID	PFBS	PFHxS	PFOS	IA PFOS	IA PFHxS	PFBA	PFHxA	PFOA	PFNA	PFDA	PFUnDA	PFDoDA	FOSA	6:2 FTSA	8:2 FTSA
W1	71	92	78	72	87	13	46	88	80	76	69	69	57	105	106
W2	69	85	73	71	80	11	51	84	77	69	61	55	54	84	67
W3*	48	53	43	39	50	46	43	55	48	41	36	32	35	29	29
W4	77	91	61	59	87	22	45	85	73	54	50	49	52	104	100
W5	68	87	60	58	83	25	48	84	72	52	44	41	30	101	83
W6*	32	36	31	30	34	39	28	38	33	30	26	24	18	23	25
W7	79	88	61	57	84	5	49	84	69	57	56	58	30	115	154
W8	66	87	72	67	83	9	47	86	76	68	60	55	54	101	74
W9	66	80	67	65	75	67	54	78	70	63	55	53	44	66	77
S2	90	90	87	84	86	49	90	90	88	88	85	85	29	76	130
S3	82	84	81	76	80	48	59	62	64	70	74	76	20	67	123
E1	765	73	66	62	69	14	441	65	67	65	57	44	54	38	124
E2	579	52	50	48	50	3	54	55	53	51	44	26	41	130	213
E4	316	62	58	55	59	49	191	58	56	55	50	50	24	46	239
SW1	58	61	58	55	58	23	46	59	57	56	52	52	18	66	61
SW2*	38	52	49	46	49	12	29	48	47	47	47	40	2	92	78
SW3	70	94	90	86	89	36	69	93	91	91	90	89	11	151	173
SW4	109	93	93	88	86	28	76	91	92	91	89	107	22	124	219
SW5	95	93	86	84	87	6	70	86	88	87	84	76	8	116	221
SW6	82	93	87	84	89	6	70	89	89	89	87	80	7	125	265
SW7	74	87	84	80	83	27	62	84	85	86	81	65	4	154	147
SW8	80	91	89	85	86	55	68	89	89	89	88	88	14	149	175
SW9	77	72	72	68	68	28	57	70	69	69	66	70	30	84	103
SW10	115	89	86	82	85	14	70	84	86	86	81	73	12	107	192
SW11	68	67	66	62	63	35	52	66	66	66	61	51	23	63	80
SW12*	28	27	29	27	26	2	21	29	28	27	25	27	13	28	49
SW13	71	72	91	88	80	2	26	50	56	60	57	14	16	40	97
SW14	91	84	84	79	80	5	42	70	76	79	75	27	23	73	170
S1*	16	25	8	17	16	16	18	19	17	12	16	16	18	14	24
D1	96	81	80	78	77	56	84	79	78	82	76	77	40	63	214
D2	98	82	83	79	78	9	73	83	83	83	79	166	13	63	154
D3	93	79	79	75	76	54	51	58	58	59	58	56	33	32	103
D4*	80	68	67	64	65	44	53	53	52	53	53	49	24	55	198
D5	91	83	79	75	79	54	16	79	80	75	55	28	11	78	108
D6	88	77	72	70	73	58	67	70	70	71	68	63	24	40	130
D7	91	79	76	73	75	41	53	56	56	61	60	55	21	63	220
D8	88	76	74	71	73	45	53	55	56	57	56	50	24	57	191
D9	90	66	66	63	63	44	16	69	68	70	65	68	8	95	156
D10	93	79	78	75	75	69	57	78	78	77	73	69	37	46	187
D11	88	73	75	73	70	62	15	74	73	74	66	85	15	76	189

D12	92	78	73	70	74	60	61	74	75	77	69	61	28	40	162
D13	93	83	79	75	79	60	75	79	79	76	70	63	26	62	208
D14	133	83	75	72	79	58	17	77	77	77	68	51	44	38	140
D15	94	80	75	71	76	58	17	77	76	76	68	51	30	39	148
D16	93	80	74	71	76	59	65	76	76	76	69	51	32	50	191
D17	102	94	89	88	90	66	20	92	90	88	81	54	42	100	263
D18	101	95	85	82	90	48	12	65	65	66	68	55	41	98	249
D19	86	88	80	78	84	42	11	59	60	60	61	52	40	54	198
D20	107	91	87	91	86	63	15	76	77	79	70	60	49	73	199
L1	106	92	92	94	88	55	54	67	69	69	70	156	46	50	200
L2	89	86	84	82	82	76	71	85	84	87	81	156	39	105	160
L3	97	93	93	89	89	68	65	83	83	85	83	86	43	113	218
L4	110	95	94	95	90	52	72	88	90	92	89	96	44	80	167
L5	93	88	82	78	83	40	52	84	83	82	76	87	37	69	165
L6	91	91	88	85	86	15	66	89	89	88	83	63	38	122	215
L7	92	87	82	80	83	23	71	82	82	85	77	68	34	93	206
L8	96	97	93	90	92	19	71	92	91	87	87	78	41	47	165
L9	79	78	74	74	74	58	56	76	75	75	70	75	33	101	185
L10	106	92	93	90	88	72	69	87	87	91	88	103	42	106	158
L11	103	99	93	89	94	51	71	93	94	94	89	127	38	91	152
L12	82	101	94	89	96	35	63	94	96	94	92	79	47	149	220
L13	131	93	90	84	88	80	90	88	96	100	88	97	40	144	247
L14	105	99	96	91	94	28	87	97	97	101	97	168	46	87	143
L15	95	89	88	84	84	48	69	85	86	87	81	69	15	98	137
L16	88	100	95	91	94	29	66	95	95	97	92	52	6	119	146
P1	114	95	93	88	90	70	28	94	93	93	85	36	6	125	130
P2	115	94	93	88	89	4	79	91	92	93	87	39	4	91	131
P3	48	53	53	51	50	6	38	54	53	53	50	35	16	57	107
P4	82	97	94	89	92	16	58	92	95	96	89	63	14	102	221
P5	96	95	93	89	90	8	80	94	92	92	92	75	26	51	118
P6	100	83	81	77	79	3	65	79	81	81	78	50	17	63	147
P7	601	92	81	81	88	6	98	87	86	89	85	65	22	100	128
P8	585	96	88	84	94	2	77	87	87	88	86	73	27	112	151
P9	93	92	93	89	87	9	51	72	77	79	75	49	10	66	126
P10	106	92	91	86	88	10	80	93	90	91	87	64	13	114	162
P11	100	90	89	85	86	5	65	84	84	87	83	76	10	95	139
P12	65	93	94	90	89	13	47	94	94	94	88	50	14	138	199
P13	68	97	94	90	92	13	21	96	94	98	92	43	8	139	196
P14	66	85	86	82	81	27	51	85	84	87	79	52	21	129	191
P15	78	95	90	85	90	5	19	88	86	86	87	39	5	131	164
P16	75	91	89	85	87	8	21	88	87	89	85	44	12	126	190
P17	79	94	94	90	89	17	21	92	91	92	87	49	13	135	218
P18	96	90	86	82	86	24	81	85	88	87	83	60	21	90	211

P19	91	80	81	77	76	26	87	80	78	82	75	62	40	58	168
P20	59	67	68	65	63	15	49	67	67	67	63	45	30	80	181
P21	78	91	91	87	86	19	52	89	89	89	86	73	31	86	196
P22	101	90	89	85	86	6	87	87	88	87	86	82	41	77	264
P23	111	92	91	87	88	4	84	90	90	89	87	72	42	73	191
P24	106	92	91	87	88	3	76	89	88	93	85	63	43	64	150
P25	143	92	90	86	87	2	64	87	88	89	82	67	24	94	140
P26	80	84	86	82	81	1	27	69	81	84	82	72	21	73	165
P27	637	96	85	81	90	67	54	92	87	84	76	59	20	131	159
P29	111	91	86	81	87	24	76	86	85	83	74	85	62	87	203
P30	203	81	76	72	76	63	19	74	74	75	72	78	52	153	234
P31	108	91	87	83	87	72	24	90	89	87	85	31	3	142	132
P32	77	92	86	83	88	2	56	90	88	88	83	45	10	97	163
P33	90	95	95	90	91	12	54	94	94	96	92	41	7	104	138
P34	99	88	86	82	83	4	84	87	87	89	86	76	49	81	300
P35	462	97	93	88	89	3	93	93	96	93	88	74	37	192	273
P36	119	93	96	90	88	3	82	94	92	91	89	88	24	116	204
D21	116	91	93	88	87	2	58	89	91	92	90	70	12	75	122
D22	114	91	94	89	87	11	79	90	90	91	88	77	25	65	130
D23	113	90	88	81	86	5	73	87	87	88	86	69	13	83	118
D24*	36	30	32	30	28	10	29	32	31	30	28	24	12	19	77
D25*	45	38	35	34	36	30	28	36	35	33	30	31	29	20	62
D26	105	89	87	82	84	64	65	87	87	88	81	54	16	56	109
D27	105	85	88	84	81	10	74	86	87	89	87	40	8	42	101
D28	110	87	87	83	83	2	69	85	85	85	83	44	8	55	79

*samples with lower recoveries due to losses during the extraction, however quantified concentrations were recovery corrected.

Table S7. Concentrations of all PFSAs and branched PFOS and PFHxS isomers in all samples (ng L^{-1} for water and ng g^{-1} dw for all other). Concentration below the method detection limited are marked with <MDL.

ID	PFBS	PFPeS	∑PFHxS	PFHpS	∑PFOS	PFNS	PFDS	PFDoDS	1m-PFOS	6/2m-PFOS	3/4/5m-PFOS	1m- PFHxS	2/4m- PFHxS	3m- PFHxS
W1	81.8	6.4	58.4	4.6	228.6	0.3	<MDL	<MDL	9.9	37.2	52.1	0.7	7.7	2.5
W2	39.5	3.1	27.9	2.6	140.0	0.1	<MDL	<MDL	6.9	22.6	34.1	0.5	3.8	1.2
W3	0.1	<MDL	0.1	<MDL	0.1	<MDL	<MDL	<MDL	<MDL	<MDL	<MDL	<MDL	<MDL	<MDL
W4	129.0	24.1	211.7	25.8	1044.6	1.0	0.2	<MDL	52.7	186.8	260.8	2.7	28.7	9.2
W5	43.3	3.6	34.9	5.7	442.2	0.4	<MDL	<MDL	24.6	76.3	109.8	0.6	4.4	1.5
W6	0.1	<MDL	0.1	<MDL	0.8	<MDL	<MDL	<MDL	<MDL	0.1	0.2	<MDL	<MDL	<MDL
W7	174.8	15.7	138.4	13.4	539.7	0.6	0.1	<MDL	26.7	93.7	135.9	1.9	19.3	5.6
W8	23.8	1.8	16.6	1.4	54.9	<MDL	<MDL	<MDL	2.7	9.1	13.4	0.2	2.2	0.7
W9	0.1	<MDL	0.1	<MDL	0.5	<MDL	<MDL	<MDL	<MDL	0.1	0.1	<MDL	<MDL	<MDL
L1	<MDL	<MDL	2.4	2.1	486.1	1.5	3.0	<MDL	14.2	30.1	34.0	<MDL	<MDL	<MDL
L2	18.9	1.6	22.5	1.7	448.2	3.2	2.1	<MDL	7.6	17.8	24.2	<MDL	1.2	0.8
L3	0.7	0.6	17.4	2.2	546.7	4.3	2.1	<MDL	5.2	12.6	12.5	<MDL	0.3	0.3
L4	3.3	0.8	8.0	1.4	334.2	2.1	1.7	<MDL	5.3	7.8	9.4	<MDL	1.3	0.6
L5	1.4	0.8	17.0	2.1	310.7	2.6	2.4	0.7	3.9	8.6	11.9	0.3	1.1	0.8
L6	0.2	0.2	28.0	1.6	188.1	2.2	2.2	0.3	2.4	5.9	7.5	<MDL	0.7	0.2
L7	0.3	0.2	15.6	1.1	194.8	2.0	2.0	0.7	2.0	5.3	5.1	<MDL	0.3	<MDL
L8	0.4	0.2	17.5	1.2	214.6	2.3	2.0	0.4	1.9	6.2	6.2	<MDL	0.5	0.2
L9	1.7	1.0	41.5	9.2	1991.0	6.2	4.9	2.0	32.4	124.2	124.4	<MDL	1.3	0.5
L10	2.2	1.3	29.5	6.1	1148.0	4.8	5.9	3.4	14.8	41.7	46.2	<MDL	1.3	0.5
L11	1.0	0.6	26.8	4.8	747.8	3.4	3.6	2.2	8.9	31.4	<MDL	<MDL	1.0	0.6
L12	0.4	0.3	24.0	9.0	1651.6	4.8	3.1	2.1	48.4	165.7	180.1	<MDL	0.7	0.5
L13	0.3	<MDL	0.7	<MDL	0.9	<MDL	<MDL	<MDL	<MDL	<MDL	<MDL	<MDL	0.4	<MDL
L14	14.7	<MDL	<MDL	<MDL	1.6	<MDL	<MDL	<MDL	0.1	<MDL	0.1	<MDL	<MDL	<MDL
L15	0.3	<MDL	<MDL	<MDL	2.3	<MDL	<MDL	<MDL	0.1	<MDL	0.1	<MDL	<MDL	<MDL
L16	0.1	<MDL	<MDL	<MDL	2.1	<MDL	<MDL	<MDL	0.1	<MDL	0.1	<MDL	<MDL	<MDL
E1	1.6	20.2	82.2	3.4	976.1	4.0	4.8	13.0	2.4	9.1	2.7	<MDL	<MDL	<MDL
E2	1.1	20.9	51.5	2.8	558.8	1.5	0.7	<MDL	2.9	6.6	2.4	<MDL	<MDL	<MDL
E4	0.6	4.3	6.2	<MDL	29.6	<MDL	<MDL	<MDL	<MDL	1.0	<MDL	<MDL	<MDL	<MDL
SW1	1.5	0.9	44.6	6.4	830.2	3.5	3.1	0.9	10.1	43.5	44.5	0.2	1.8	0.4
SW2	0.2	<MDL	5.2	1.0	162.1	0.6	0.5	0.2	1.6	6.2	4.9	<MDL	<MDL	<MDL
SW3	4.8	0.5	7.5	0.7	94.9	0.4	0.5	0.3	0.8	2.8	2.8	<MDL	0.4	<MDL
SW4	0.2	<MDL	1.8	0.3	71.5	0.5	0.2	<MDL	0.8	1.6	1.3	<MDL	<MDL	<MDL
SW5	<MDL	<MDL	2.8	0.5	140.9	0.9	0.4	<MDL	1.5	3.1	2.3	<MDL	<MDL	<MDL
SW6	<MDL	<MDL	1.8	0.3	92.7	0.6	0.3	<MDL	0.8	2.0	1.6	<MDL	<MDL	<MDL
SW7	0.2	0.2	12.4	0.9	107.5	0.6	0.5	0.2	0.9	3.3	3.3	<MDL	0.3	<MDL
SW8	0.1	<MDL	4.7	0.6	54.8	0.4	0.3	<MDL	0.4	2.4	1.5	<MDL	<MDL	<MDL
SW9	0.1	<MDL	5.9	0.5	136.4	0.9	0.5	<MDL	0.8	3.6	2.7	<MDL	<MDL	<MDL
SW10	<MDL	<MDL	<MDL	<MDL	<MDL	<MDL	<MDL	<MDL	<MDL	<MDL	<MDL	<MDL	<MDL	<MDL
SW11	0.1	<MDL	0.2	<MDL	3.0	<MDL	<MDL	<MDL	<MDL	<MDL	<MDL	<MDL	<MDL	<MDL

SW12	<MDL	<MDL	0.3	<MDL	1.9	<MDL	<MDL	<MDL	<MDL	0.3	<MDL	<MDL	<MDL	<MDL
SW13	0.0	<MDL	<MDL	<MDL	6.2	<MDL	<MDL	<MDL	<MDL	0.4	0.3	<MDL	<MDL	<MDL
SW14	0.1	<MDL	<MDL	<MDL	5.7	<MDL	<MDL	<MDL	<MDL	0.4	0.4	<MDL	<MDL	<MDL
D1	2.2	1.6	41.3	4.4	492.4	1.5	1.8	<MDL	16.9	46.3	51.7	0.3	2.0	0.6
D2	0.2	<MDL	1.6	<MDL	67.8	0.3	0.9	<MDL	1.8	3.5	4.1	<MDL	<MDL	<MDL
D3	0.1	<MDL	0.3	<MDL	69.0	0.6	0.3	<MDL	1.4	1.6	1.4	<MDL	<MDL	<MDL
D4	<MDL	<MDL	0.2	<MDL	19.2	<MDL	<MDL	<MDL	0.9	1.2	1.5	<MDL	<MDL	<MDL
D5	<MDL	<MDL	0.1	<MDL	11.5	0.1	<MDL	<MDL	0.2	0.5	0.3	<MDL	<MDL	<MDL
D6	0.1	<MDL	0.2	<MDL	22.4	0.2	<MDL	<MDL	0.7	1.1	1.3	<MDL	<MDL	<MDL
D7	0.0	<MDL	0.2	<MDL	25.7	0.3	<MDL	<MDL	0.9	1.3	1.4	<MDL	<MDL	<MDL
D8	0.1	<MDL	0.3	<MDL	12.5	0.1	<MDL	<MDL	0.4	0.5	0.6	<MDL	<MDL	<MDL
D9	0.1	<MDL	0.9	0.3	100.8	0.4	0.2	<MDL	4.4	6.3	8.8	<MDL	<MDL	<MDL
D10	<MDL	<MDL	0.1	<MDL	0.8	<MDL	<MDL	<MDL	0.1	<MDL	0.1	<MDL	<MDL	<MDL
D11	<MDL	<MDL	0.2	<MDL	1.8	<MDL	<MDL	<MDL	0.1	0.2	0.1	<MDL	<MDL	<MDL
D12	0.1	<MDL	2.4	1.0	295.9	2.6	1.6	<MDL	3.4	7.4	7.3	<MDL	<MDL	<MDL
D13	0.1	<MDL	0.2	<MDL	1.8	<MDL	<MDL	<MDL	0.1	<MDL	0.1	<MDL	<MDL	<MDL
D14	1.4	3.9	59.7	2.6	469.5	4.7	2.9	<MDL	4.5	22.6	32.3	0.5	3.0	0.8
D15	1.0	3.2	56.2	2.5	398.6	4.3	2.1	<MDL	3.8	18.0	21.5	0.2	1.8	0.4
D16	1.0	4.9	70.5	4.3	741.9	7.1	3.9	<MDL	7.0	28.7	32.0	0.2	1.2	0.3
D17	0.1	<MDL	8.9	0.8	158.3	1.2	0.8	<MDL	1.3	4.2	3.8	<MDL	<MDL	<MDL
D18	0.1	<MDL	2.1	0.4	122.0	0.8	0.2	<MDL	1.8	4.6	3.9	<MDL	<MDL	<MDL
D19	0.1	<MDL	2.2	0.2	52.1	0.4	0.2	<MDL	0.5	1.8	1.2	<MDL	<MDL	<MDL
D20	0.1	<MDL	5.0	0.6	112.7	0.9	0.4	<MDL	1.0	3.3	2.7	<MDL	0.2	<MDL
D21	0.1	<MDL	0.7	0.2	102.9	1.4	0.7	<MDL	1.8	4.9	7.1	<MDL	<MDL	<MDL
D22	<MDL	<MDL	0.9	0.4	354.4	3.5	1.3	<MDL	5.6	7.4	9.3	<MDL	<MDL	<MDL
D23	<MDL	<MDL	0.8	0.3	532.0	8.4	2.7	<MDL	7.4	12.4	12.2	<MDL	<MDL	<MDL
D24	0.5	4.5	135.4	8.5	1447.4	14.7	9.4	<MDL	12.1	77.1	83.8	0.7	3.5	0.9
D25	0.3	3.4	93.3	5.5	885.8	9.8	7.4	<MDL	6.3	41.7	49.4	0.4	2.3	0.7
D26	0.1	0.2	9.6	0.9	204.5	1.1	0.5	<MDL	1.2	5.4	4.0	<MDL	0.2	<MDL
D27	0.2	0.7	11.4	3.2	1093.8	9.3	5.3	<MDL	12.8	26.5	26.0	<MDL	<MDL	<MDL
D28	0.1	<MDL	<MDL	<MDL	3.1	<MDL	<MDL	<MDL	0.5	0.2	0.2	<MDL	<MDL	<MDL
S2	0.3	0.2	3.9	0.9	223.2	0.8	0.3	0.3	7.3	17.5	22.2	<MDL	0.2	<MDL
S3	<MDL	<MDL	<MDL	<MDL	<MDL	<MDL	<MDL	<MDL	<MDL	<MDL	<MDL	<MDL	<MDL	<MDL
S1	0.8	0.6	15.7	1.3	190.0	0.4	0.5	<MDL	2.9	13.8	15.9	<MDL	0.6	0.2
P1	9.8	<MDL	4.4	1.2	236.5	0.7	2.8	<MDL	5.5	17.9	23.7	0.2	0.5	0.5
P2	3.4	0.3	5.2	1.3	77.0	0.1	0.7	<MDL	2.7	8.5	18.0	<MDL	0.2	0.2
P3	0.1	<MDL	0.8	0.2	15.2	<MDL	0.3	<MDL	0.2	0.9	0.9	<MDL	<MDL	<MDL
P4	0.3	0.1	3.8	0.8	86.2	0.2	1.5	<MDL	2.3	5.9	7.1	<MDL	0.2	<MDL
P5	0.4	0.2	5.8	1.6	245.1	0.7	2.0	0.2	7.7	17.8	20.4	<MDL	0.4	0.2
P6	0.1	<MDL	4.7	1.1	156.3	0.5	0.8	0.2	4.0	11.0	14.4	<MDL	0.4	<MDL
P7	0.4	<MDL	6.7	0.6	40.5	0.1	0.2	<MDL	0.1	<MDL	0.1	<MDL	<MDL	<MDL
P8	1.1	<MDL	8.8	2.8	399.3	2.2	2.3	1.3	0.1	36.4	0.1	<MDL	<MDL	<MDL
P9	0.6	<MDL	8.0	1.2	705.7	7.5	2.6	<MDL	18.0	38.0	49.8	<MDL	0.6	0.2

P10	1.0	1.2	14.4	1.6	635.6	6.8	3.1	<MDL	16.8	34.1	42.5	1.4	0.8	0.3
P11	1.3	0.7	11.0	1.4	618.5	5.3	2.1	<MDL	15.3	31.5	38.3	<MDL	0.5	0.2
P12	0.2	<MDL	6.7	0.5	114.9	0.6	0.3	<MDL	2.6	4.9	6.4	<MDL	<MDL	0.4
P13	<MDL	<MDL	6.9	1.3	482.5	2.8	0.9	<MDL	7.8	16.5	18.4	<MDL	0.1	0.3
P14	<MDL	0.2	7.6	0.7	176.3	1.2	0.5	<MDL	3.4	6.3	7.5	<MDL	0.2	0.5
P15	<MDL	0.4	13.3	2.5	808.3	5.7	1.4	<MDL	23.0	42.8	50.0	<MDL	<MDL	<MDL
P16	<MDL	0.2	3.2	0.5	317.9	3.6	1.4	<MDL	5.8	11.4	11.8	<MDL	<MDL	<MDL
P17	<MDL	0.3	19.2	1.9	482.7	2.5	0.9	<MDL	14.9	26.9	36.1	<MDL	<MDL	<MDL
P18	<MDL	<MDL	3.1	0.4	201.4	2.1	0.9	<MDL	4.1	9.1	11.0	<MDL	<MDL	<MDL
P19	<MDL	<MDL	1.8	0.3	164.8	1.6	0.5	<MDL	3.4	7.1	8.2	<MDL	<MDL	<MDL
P20	<MDL	<MDL	7.2	0.7	273.3	2.4	0.7	<MDL	7.5	14.3	19.1	<MDL	0.2	<MDL
P21	<MDL	<MDL	1.1	<MDL	30.9	0.2	<MDL	<MDL	0.9	1.8	2.6	<MDL	<MDL	<MDL
P22	0.1	<MDL	2.2	0.5	161.0	1.3	0.5	<MDL	4.6	9.7	12.4	<MDL	<MDL	<MDL
P23	0.1	<MDL	3.2	0.8	289.1	2.6	0.8	<MDL	8.6	17.1	22.9	<MDL	<MDL	<MDL
P24	0.1	<MDL	3.9	1.1	327.9	2.5	0.7	<MDL	10.9	20.6	27.1	<MDL	<MDL	<MDL
P25	0.2	<MDL	4.7	1.1	479.1	3.1	0.5	<MDL	10.7	25.2	<MDL	<MDL	0.3	0.2
P26	0.2	<MDL	5.1	0.8	253.2	1.3	0.3	<MDL	5.2	0.8	<MDL	<MDL	0.2	<MDL
P27	0.3	<MDL	4.9	1.0	288.0	1.4	0.3	<MDL	6.3	12.9	<MDL	<MDL	<MDL	0.3
P29	0.3	<MDL	9.3	4.4	1812.7	24.1	6.9	<MDL	32.0	133.7	117.1	<MDL	<MDL	<MDL
P30	0.9	<MDL	15.9	8.3	2777.5	41.5	10.9	<MDL	69.5	258.0	267.2	<MDL	1.4	0.5
P31	0.3	<MDL	<MDL	<MDL	19.1	<MDL	<MDL	<MDL	0.4	1.2	1.3	<MDL	<MDL	<MDL
P32	<MDL	<MDL	1.1	0.3	16.7	<MDL	<MDL	<MDL	<MDL	0.8	0.6	<MDL	<MDL	<MDL
P33	<MDL	<MDL	0.4	<MDL	11.0	<MDL	<MDL	<MDL	<MDL	0.5	0.4	<MDL	<MDL	<MDL
P34	0.1	<MDL	0.2	<MDL	2.6	<MDL	<MDL	<MDL	<MDL	0.3	0.2	<MDL	<MDL	<MDL
P35	0.1	<MDL	<MDL	<MDL	1.3	<MDL	<MDL	<MDL	<MDL	<MDL	<MDL	<MDL	<MDL	<MDL
P36	<MDL	<MDL	<MDL	<MDL	0.5	<MDL	<MDL	<MDL	<MDL	<MDL	0.0	<MDL	<MDL	<MDL

Table S8. Concentrations of target PFCAs, FOSA,FTSAs and $\sum_{24}$PFAS in all samples (ng L^{-1} for water and ng g^{-1} dw for all other). Concentration below the method detection limited are marked with <MDL. Excluded were PFTDA, PFHxDA, PFOcDA and 4:2 FTSA since they were <MDL in all samples.

ID	PFHxA	PFHpA	PFOA	PFNA	PFDA	PFUnDA	PFDoDA	PFTrDA	FOSA	6:2 FTSA	8:2 FTSA	10:2 FTSA	$\sum_{24}$PFAS
W1	56.5	25.8	21.7	7.7	2.0	0.8	0.1	<MDL	5.3	29.5	1.5	0.1	641.3
W2	26.2	14.3	8.4	3.1	0.4	0.1	<MDL	<MDL	0.5	2.8	0.1	<MDL	338.4
W3	0.2	0.2	0.2	<MDL	<MDL	<MDL	<MDL	<MDL	<MDL	<MDL	<MDL	<MDL	1.8
W4	131.2	57.0	53.9	11.2	4.6	2.1	0.3	<MDL	13.1	35.2	2.2	0.2	2288.3
W5	37.8	14.6	12.0	6.2	1.6	0.3	<MDL	<MDL	2.8	18.7	0.6	<MDL	842.1
W6	<MDL	0.2	0.2	<MDL	<MDL	<MDL	<MDL	<MDL	0.0	<MDL	<MDL	<MDL	2.5
W7	85.2	40.7	32.4	6.8	3.3	1.1	0.3	<MDL	5.6	112.8	11.9	0.2	1466.3
W8	12.3	4.9	3.9	0.7	0.2	<MDL	<MDL	<MDL	0.4	9.3	0.4	<MDL	159.2
W9	0.3	0.2	0.2	<MDL	<MDL	<MDL	<MDL	<MDL	0.0	<MDL	<MDL	<MDL	2.3
L1	15.9	<MDL	1.3	1.4	2.0	1.6	0.4	0.2	37.5	18.0	32.4	0.4	684.9
L2	11.1	<MDL	3.0	1.5	2.3	3.1	0.8	0.7	21.3	16.5	13.7	0.3	624.1
L3	2.7	<MDL	1.4	2.4	3.3	4.6	1.3	0.8	33.0	6.2	25.4	0.2	686.5
L4	<MDL	<MDL	2.2	2.8	3.5	7.2	3.3	1.2	72.8	8.2	10.9	0.2	488.5
L5	<MDL	<MDL	3.1	2.7	2.8	6.8	3.3	2.4	41.3	10.2	12.3	0.3	449.7
L6	0.7	0.3	2.2	2.3	3.1	9.2	5.3	1.5	69.5	2.8	50.1	<MDL	386.7
L7	0.6	0.3	1.9	2.2	2.5	6.6	3.7	1.8	39.0	3.3	28.0	<MDL	319.6
L8	0.7	0.3	1.6	1.8	2.3	5.0	2.8	1.5	42.1	2.9	28.2	<MDL	342.8
L9	3.6	0.7	3.1	5.4	11.4	15.1	9.7	3.4	51.1	99.7	148.3	3.2	2695.2
L10	2.4	0.6	4.3	4.9	9.1	21.5	20.8	4.6	89.7	75.2	104.2	3.5	1646.3
L11	2.4	<MDL	6.6	3.8	5.8	12.4	9.6	2.4	66.2	65.4	105.4	2.9	1115.2
L12	2.0	0.4	1.3	5.4	9.9	8.4	5.0	2.9	80.9	22.2	96.2	0.8	2326.1
L13	2.2	<MDL	<MDL	<MDL	<MDL	<MDL	0.2	0.2	0.3	0.4	<MDL	<MDL	6.5
L14	<MDL	<MDL	0.3	0.2	0.2	0.5	0.4	0.5	0.3	1.4	<MDL	<MDL	23.9
L15	<MDL	<MDL	0.2	0.3	0.3	0.5	0.3	0.3	0.5	0.5	<MDL	<MDL	8.7
L16	<MDL	<MDL	0.1	0.3	0.3	0.9	0.5	0.4	0.3	<MDL	<MDL	<MDL	8.1
E1	7.2	<MDL	3.5	2.1	3.3	3.1	5.8	13.8	1.6	5.0	2.2	1.5	1168.6
E2	2.4	<MDL	2.0	1.2	1.9	2.0	3.4	3.9	1.8	3.2	9.9	0.5	681.7
E4	2.3	<MDL	4.7	3.0	1.3	2.0	2.0	3.8	0.3	0.3	0.2	<MDL	62.1
SW1	<MDL	1.5	1.5	1.4	2.1	3.0	2.6	0.8	10.3	89.1	46.0	1.8	1151.7
SW2	2.1	<MDL	0.6	0.9	3.3	2.3	2.7	0.6	4.6	7.2	9.3	0.7	217.1
SW3	1.3	0.3	1.2	1.0	1.0	1.0	0.8	0.1	44.3	27.6	20.3	0.4	215.8
SW4	<MDL	<MDL	<MDL	0.4	0.3	0.6	<MDL	<MDL	4.1	2.8	4.4	<MDL	91.3
SW5	<MDL	<MDL	<MDL	0.6	0.6	0.7	<MDL	<MDL	8.2	3.4	7.3	<MDL	173.8
SW6	<MDL	<MDL	<MDL	0.5	0.4	1.9	<MDL	<MDL	6.0	3.5	6.1	<MDL	119.1
SW7	<MDL	<MDL	0.7	0.3	0.2	0.5	0.5	0.2	13.5	2.7	7.7	<MDL	156.8
SW8	<MDL	<MDL	0.2	0.1	0.2	0.4	0.2	<MDL	1.7	1.0	3.2	<MDL	72.6
SW9	<MDL	<MDL	0.4	0.7	0.5	0.5	0.1	<MDL	5.0	4.2	8.3	0.2	171.7
SW10	<MDL	<MDL	0.2	<MDL	<MDL	0.2	<MDL	<MDL	0.7	<MDL	<MDL	<MDL	2.9
SW11	<MDL	<MDL	0.6	0.3	0.2	0.4	<MDL	<MDL	0.3	0.3	<MDL	<MDL	6.5

SW12	<MDL	<MDL	1.2	0.9	0.5	0.5	0.3	<MDL	0.2	1.1	<MDL	<MDL	8.1
SW13	<MDL	<MDL	0.3	0.3	0.2	0.4	0.2	0.2	0.4	1.4	0.2	<MDL	11.3
SW14	<MDL	<MDL	0.3	0.4	0.4	0.3	0.2	<MDL	0.5	<MDL	<MDL	<MDL	9.7
D1	5.8	0.8	3.4	0.4	0.4	0.7	0.3	<MDL	6.6	8.7	2.6	0.3	693.1
D2	<MDL	0.3	1.8	0.7	4.5	2.7	3.5	3.2	30.0	23.7	5.0	0.3	156.6
D3	<MDL	0.1	0.7	<MDL	0.2	0.4	0.2	0.1	3.9	3.6	2.5	<MDL	86.9
D4	<MDL	0.0	0.5	<MDL	0.1	0.1	<MDL	<MDL	1.3	0.8	0.5	<MDL	52.2
D5	<MDL	<MDL	<MDL	<MDL	<MDL	<MDL	<MDL	<MDL	0.7	0.6	0.4	<MDL	15.3
D6	<MDL	0.1	0.5	<MDL	0.1	0.2	<MDL	<MDL	1.9	2.2	1.1	<MDL	33.0
D7	<MDL	0.1	0.7	<MDL	0.2	0.3	<MDL	<MDL	3.6	4.0	2.2	<MDL	41.8
D8	<MDL	0.1	0.7	<MDL	0.1	0.2	<MDL	<MDL	2.9	7.2	2.9	<MDL	29.5
D9	<MDL	<MDL	0.4	0.3	0.3	0.3	<MDL	<MDL	3.7	1.6	3.5	<MDL	132.8
D10	<MDL	<MDL	0.3	<MDL	0.1	0.1	<MDL	<MDL	0.7	0.4	<MDL	<MDL	3.6
D11	<MDL	<MDL	0.2	<MDL	0.1	0.1	<MDL	<MDL	0.7	0.2	<MDL	<MDL	4.6
D12	<MDL	0.1	0.4	<MDL	0.5	1.1	0.4	0.2	4.4	1.4	5.2	0.1	335.8
D13	<MDL	0.1	0.7	<MDL	0.3	0.6	0.3	0.3	1.3	0.5	<MDL	<MDL	7.0
D14	<MDL	2.5	3.2	1.1	0.8	1.5	0.7	0.3	9.8	2.8	14.9	0.1	646.3
D15	<MDL	1.9	3.6	1.1	0.8	1.5	0.5	0.3	6.2	2.9	11.7	0.2	544.2
D16	0.3	0.8	3.8	1.6	1.3	2.5	0.9	0.5	9.1	3.7	22.5	0.2	950.3
D17	<MDL	<MDL	0.4	0.4	0.4	0.6	0.2	<MDL	13.9	2.6	10.2	<MDL	208.6
D18	<MDL	<MDL	0.2	0.4	0.4	0.2	<MDL	<MDL	5.2	3.6	6.1	<MDL	152.6
D19	<MDL	<MDL	0.2	0.2	0.2	0.2	<MDL	<MDL	3.8	3.1	6.0	<MDL	73.0
D20	<MDL	<MDL	0.3	0.3	0.3	0.4	<MDL	<MDL	6.6	3.4	9.5	<MDL	148.3
D21	<MDL	<MDL	0.2	0.4	0.2	0.5	0.3	<MDL	3.4	4.5	12.7	<MDL	142.8
D22	<MDL	<MDL	0.2	0.4	0.2	0.6	0.3	<MDL	4.4	6.9	16.9	0.2	413.3
D23	<MDL	<MDL	0.2	0.2	0.4	1.7	0.5	<MDL	8.6	2.6	13.3	0.2	604.6
D24	<MDL	0.4	7.6	1.9	1.9	3.2	1.2	0.6	19.7	5.9	43.9	0.4	1885.4
D25	<MDL	0.4	6.3	1.5	1.3	2.4	1.1	0.8	12.9	5.5	38.5	0.4	1177.6
D26	<MDL	<MDL	0.4	0.4	0.5	0.6	0.2	<MDL	9.9	1.0	7.9	<MDL	249.2
D27	<MDL	<MDL	0.6	0.2	0.6	1.5	0.8	<MDL	34.7	2.1	7.8	<MDL	1238.0
D28	<MDL	<MDL	<MDL	<MDL	<MDL	<MDL	<MDL	<MDL	1.6	0.4	<MDL	<MDL	7.2
S2	1.1	0.2	0.7	<MDL	<MDL	0.1	<MDL	<MDL	3.2	0.4	0.2	<MDL	283.4
S3	<MDL	<MDL	<MDL	<MDL	<MDL	<MDL	<MDL	<MDL	<MDL	<MDL	<MDL	<MDL	2.0
S1	2.0	0.3	1.0	0.2	0.2	0.3	<MDL	<MDL	2.1	2.7	0.8	0.2	218.6
P1	<MDL	1.1	8.3	5.6	43.3	12.9	17.9	8.2	20.8	7.9	10.9	0.5	635.8
P2	<MDL	0.3	13.1	13.0	77.6	17.9	26.4	8.2	6.6	8.9	3.2	<MDL	346.9
P3	<MDL	0.0	0.7	0.3	1.8	0.3	0.5	0.6	0.2	<MDL	0.4	<MDL	37.3
P4	<MDL	0.2	6.0	2.4	10.9	2.5	3.2	2.2	0.7	0.6	0.5	0.1	209.2
P5	<MDL	<MDL	3.0	4.3	22.0	3.5	4.5	3.2	0.7	0.7	0.5	<MDL	544.4
P6	0.1	0.1	1.5	1.7	7.8	1.9	1.7	0.7	0.5	1.1	2.6	<MDL	340.6
P7	0.7	<MDL	32.2	10.1	22.0	3.0	3.5	3.7	0.2	2.8	0.4	<MDL	170.3
P8	2.1	<MDL	20.0	8.4	32.3	10.4	11.0	9.8	0.6	6.6	9.2	<MDL	931.1
P9	0.6	1.0	1.4	2.3	2.5	3.5	1.0	0.4	28.3	7.1	25.2	<MDL	1510.5

P10	<MDL	0.6	2.3	4.0	2.8	4.4	1.9	0.8	15.6	3.6	25.3	0.2	1366.2
P11	0.6	0.4	1.2	2.2	1.7	2.7	0.2	0.4	17.3	5.7	20.3	0.1	1316.5
P12	<MDL	<MDL	0.3	0.6	0.4	0.5	<MDL	0.2	2.3	1.3	2.7	<MDL	247.8
P13	<MDL	<MDL	0.4	1.3	0.9	0.9	<MDL	0.2	7.5	2.5	9.1	<MDL	1002.2
P14	<MDL	<MDL	0.3	0.7	0.5	0.6	<MDL	0.2	3.8	1.6	4.4	<MDL	376.9
P15	<MDL	4.5	5.7	10.1	5.1	5.3	1.8	0.4	12.2	4.9	3.3	<MDL	1001.3
P16	<MDL	<MDL	0.5	1.1	0.7	1.4	0.4	0.3	1.5	1.0	1.9	<MDL	365.1
P17	<MDL	2.9	3.7	6.3	1.9	1.7	0.4	0.2	6.3	3.6	2.7	<MDL	615.6
P18	<MDL	0.4	0.6	0.9	0.7	1.1	0.3	0.2	2.2	1.1	3.9	<MDL	243.9
P19	<MDL	0.1	0.3	0.6	0.5	0.7	0.2	0.2	1.3	1.6	1.8	<MDL	195.4
P20	<MDL	1.0	1.9	1.9	0.7	0.8	0.2	0.2	2.6	2.6	2.4	<MDL	340.2
P21	<MDL	<MDL	0.2	0.4	0.3	0.4	0.2	0.3	1.1	1.7	1.4	<MDL	44.0
P22	<MDL	0.2	0.5	1.1	0.8	0.7	0.2	<MDL	0.9	1.4	4.4	<MDL	203.0
P23	<MDL	0.1	1.1	2.3	1.0	1.2	0.3	<MDL	1.5	1.7	11.8	<MDL	366.8
P24	<MDL	0.1	0.9	2.5	1.0	1.1	0.3	<MDL	1.5	1.3	12.0	<MDL	416.0
P25	1.0	0.4	6.7	12.7	6.1	6.3	1.4	0.4	7.7	7.7	7.9	<MDL	583.7
P26	0.6	0.5	5.3	8.1	3.5	3.5	0.8	0.4	1.9	3.1	2.3	<MDL	297.5
P27	1.0	0.4	6.0	9.9	3.8	3.6	1.0	0.4	2.3	2.5	1.6	<MDL	348.1
P29	2.5	1.5	11.0	24.3	14.3	20.9	5.6	3.5	2.9	6.5	9.7	<MDL	398.3
P30	<MDL	22.6	14.8	35.5	21.4	27.0	6.0	2.8	3.7	7.3	20.1	<MDL	3613.3
P31	<MDL	0.6	0.5	1.5	1.5	2.9	1.7	1.4	0.9	0.4	0.1	<MDL	34.5
P32	<MDL	<MDL	0.2	0.4	0.3	0.5	0.2	0.3	0.3	0.3	0.2	<MDL	22.8
P33	<MDL	<MDL	0.4	0.6	0.4	0.7	0.4	0.4	<MDL	0.2	<MDL	<MDL	16.2
P34	<MDL	<MDL	0.2	<MDL	0.3	0.3	<MDL	<MDL	0.3	1.4	<MDL	<MDL	6.9
P35	<MDL	<MDL	1.4	2.2	0.7	0.8	0.2	0.3	<MDL	0.2	<MDL	<MDL	8.2
P36	<MDL	<MDL	<MDL	<MDL	<MDL	0.2	<MDL	<MDL	<MDL	<MDL	<MDL	<MDL	2.1

Table S9. Emergent aquatic insect number, biomass, and insect-mediated transfer (m^{-2} d^{-1}) based on insect caught in land from four periods (T1-T4) during 2018 at the lake (KS).

KS	Date deployment	Date collection	Number of days	Number of individuals (m^{-2} d^{-1})	Biomass (mg dw m^{-2} d^{-1})	PFAS transfer (ng m^{-2} d^{-1})
T1	20180502	20180515	13	39	11	1.8
T2	20180515	20180528	13	55	34	91
T3	20180528	20180612	16	87	23	19
T4	20180612	20180703	21	52	19	2.5
Total	20180502	20180703	63	233	93	113

Table S10. Comparison of emergent insect deposition/transfer of PFOA and PFOS (ng m^{-2} d^{-1}) and estimated PFOS deposition for the whole emergence period (63 days) at the lake (KS), the stream (K1) and the reference pond (KRef) to wet deposition values found in literature.

Deposition	ng PFOA m^{-2} d^{-1}	ng PFOS m^{-2} d^{-1}	Emergence period (63 days) ng PFOS m^{-2}
Emergent insect deposition KS 2017	0.76	246.4	15524
Emergent insect deposition KS 2018	0.12	18.8	1184
Emergent insect deposition K1 2018	2.1	2.1	134
Emergent insect deposition KRef 2017	0.36	0.36	23
Emergent insect deposition KRef 2018	0.01	0.05	3
Rain deposition at a semi-rural area in Germany[8]	<blk-13.9	0.1-11.9	
Rain deposition in 28 cities in China[9]	3.9-1800	10-510	
Rain + snow deposition in northern Sweden in 2011/2012 (C2)[10]	0.083	0.057	
Rain deposition in Råö, Sweden (2015)[11]	0.53-1.15	1.2-7.4	
Rain deposition in Stockholm, Sweden (2015)[11]	0.33-5.2	0.09-3.0	

Table S11. Results from Dunn post hoc test on differences in $\delta^{15}N$, $\delta^{13}C$ and $\sum_{24}$PFAS among sample groups from sites K1 and KS. Different small letters indicate significant differences ($p<0.05$).

**Site**	**Group**	**$\delta^{15}N$**	**$\delta^{13}C$**	**$\sum_{24}$PFAS**
KS	Aquatic insect larvae (*n* = 10)	a	a	a
	Emergent aquatic insects (*n* = 8)	ab	ab	b
	Terrestrial consumers (*n* = 19)	b	b	a
K1	Aquatic insect larvae (*n* = 7)	a	a	a
	Emergent aquatic insects (*n* = 3)	ab	ab	ab
	Terrestrial consumers (*n* = 8)	b	b	b

Figures

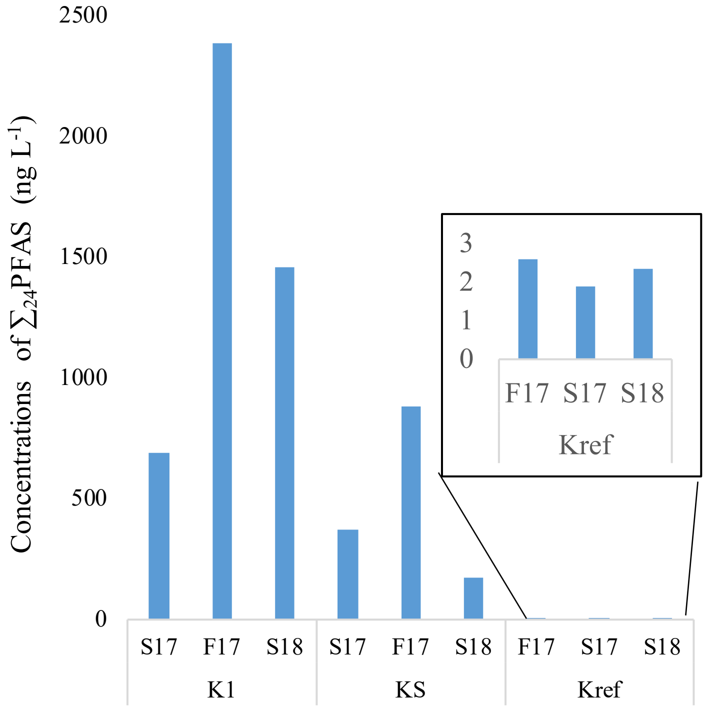

Figure S1. PFAS surface water concentrations (ng L^{-1} $\sum_{24}$PFAS) in spring 2017 (S17), fall 2017 (F17), and spring 2018 (S18) at the stream (K1), the lake (KS), and the reference pond (KRef).

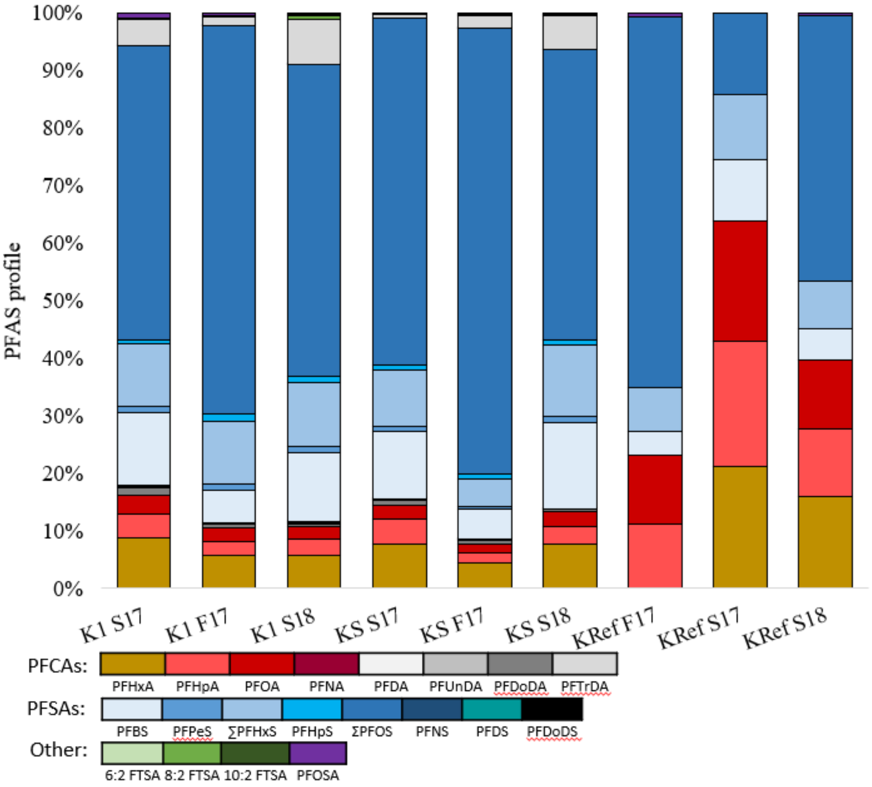

Figure S2. PFAS profiles of surface water samples from the stream (K1), the lake (KS) and the reference pond (KRef) sampled in spring 2017 (S17), fall 2017 (F17) and spring 2018 (S18).

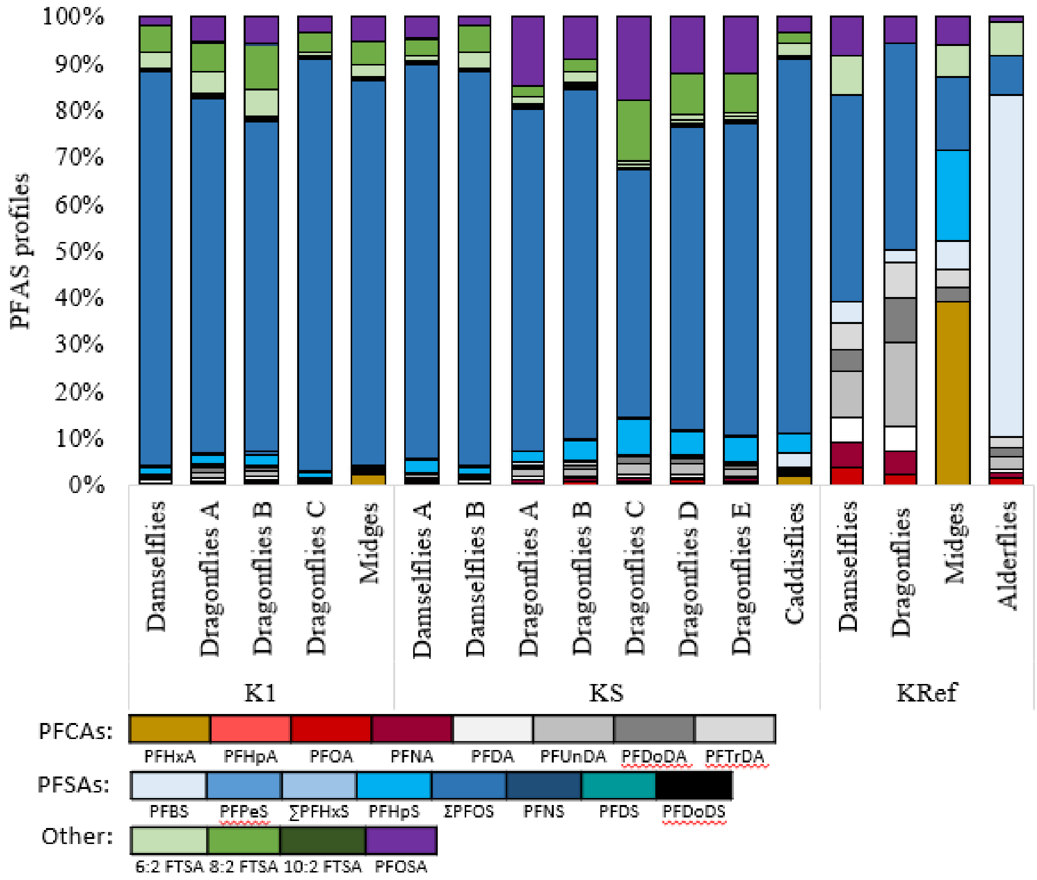

Figure S3. PFAS profiles of pooled aquatic insect larvae samples from sites K1, KS and KRef.

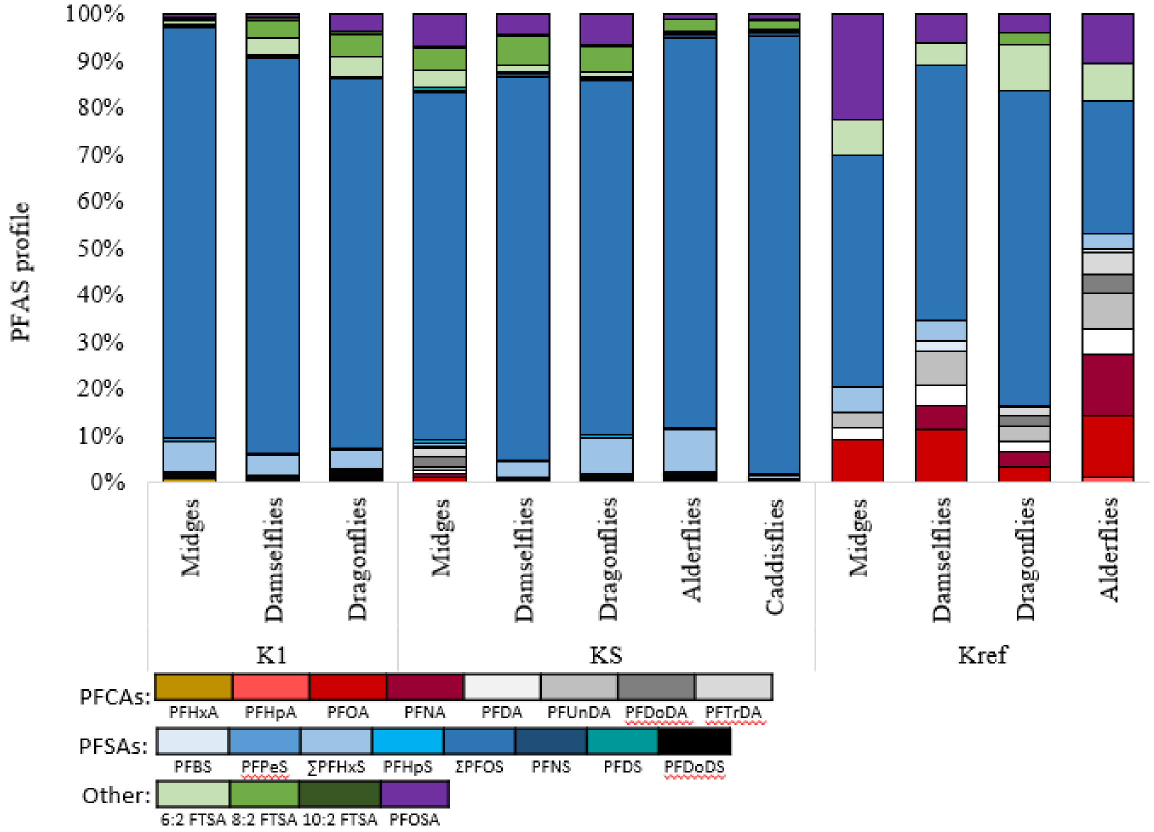

Figure S4. PFAS profiles of pooled emergent aquatic insect samples from sites K1, KS and KRef.

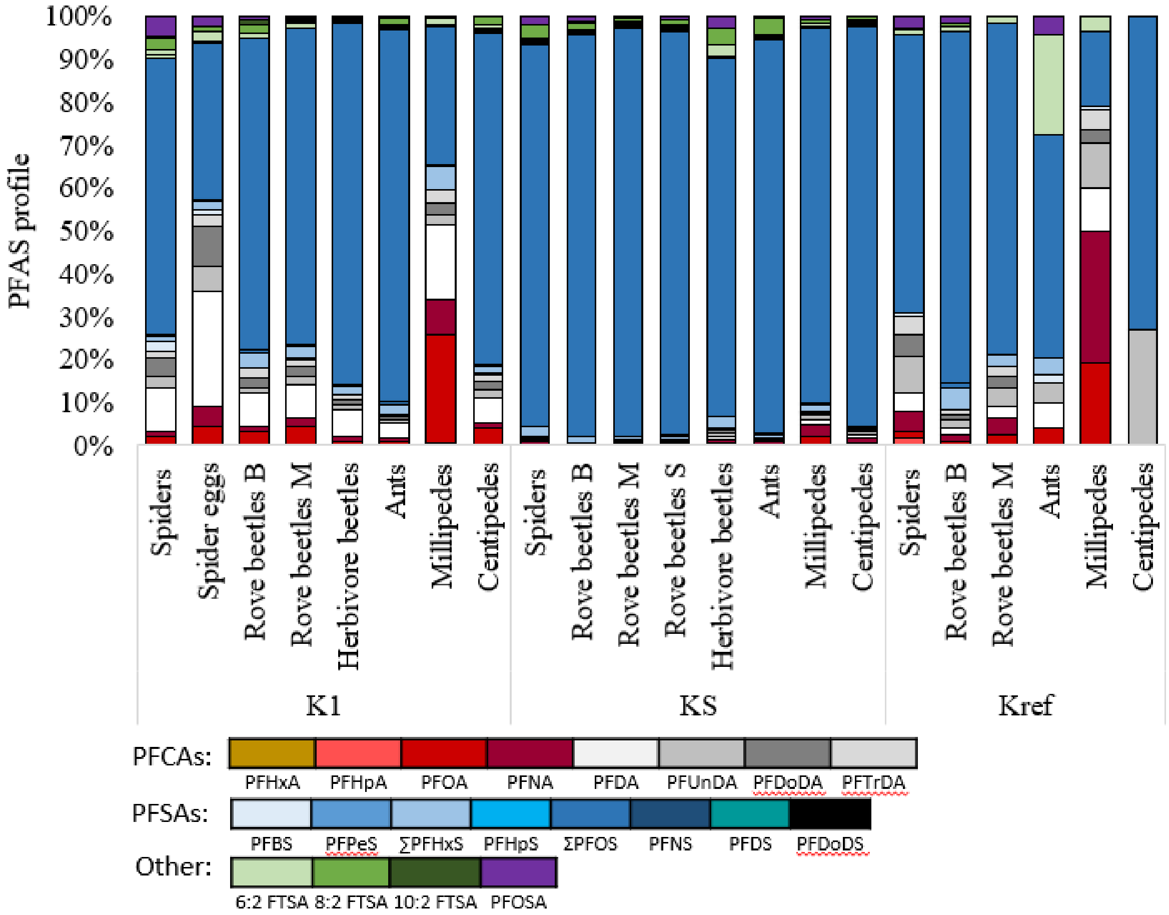

Figure S5. PFAS profiles of pooled terrestrial invertebrate consumer samples from sites K1, KS and KRef.

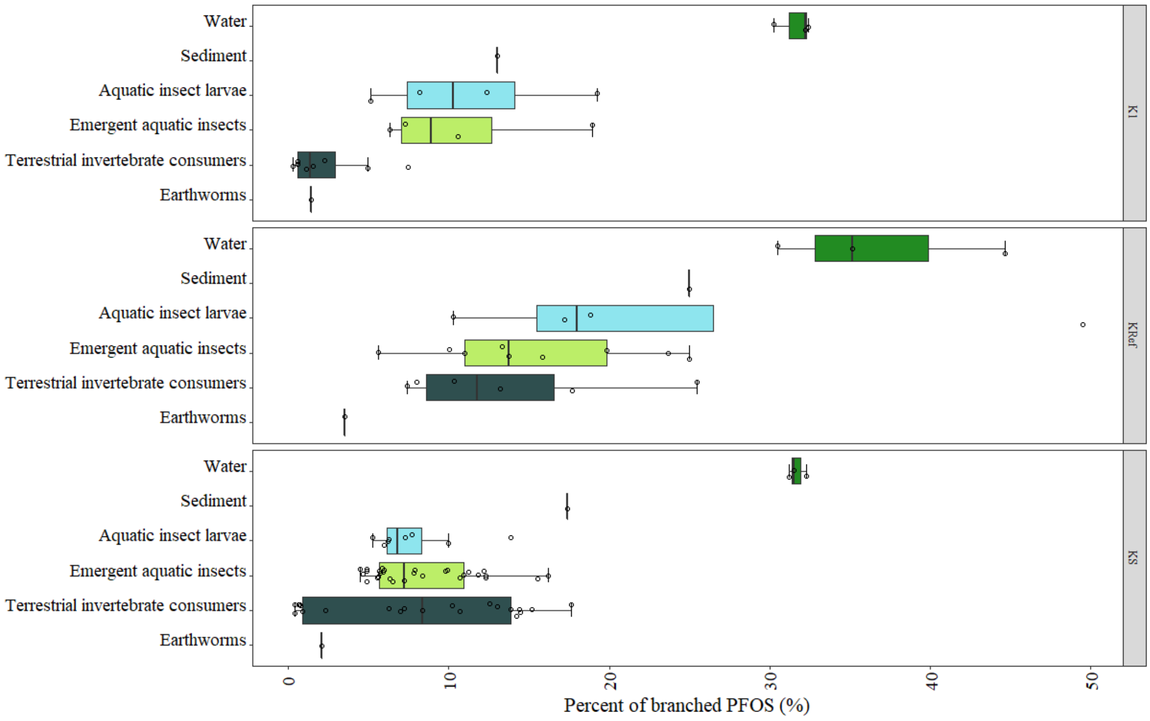

Figure S6. Percentage contribution of branched PFOS isomers to the total concentration of PFOS in each group from the stream (K1), the lake (KS), and the reference pond (KRef). Circles represent data points and the band represent the median. Lower and upper hinges correspond to the first and third quantile (25th and 75th, respectively). The lower and upper whiskers extend from the hinge to the smallest and the larges value no further than 1.5 times the inter-quartile range (IQR).

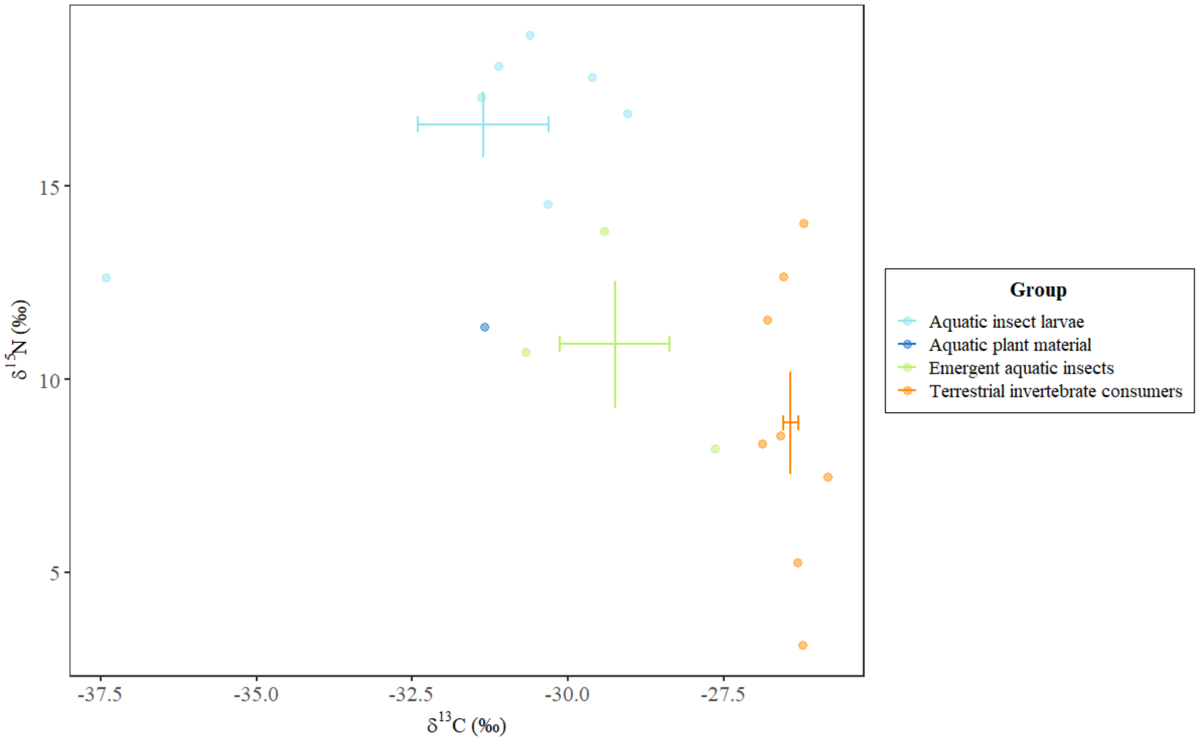

Figure S7. Values (± 1 SE) of ^{13}C vs. ^{15}N for aquatic insect larvae (light blue circles), emergent aquatic insects (green circles), aquatic plant material (dark blue circle) and terrestrial invertebrate consumers (orange circles) collected from the stream (K1).

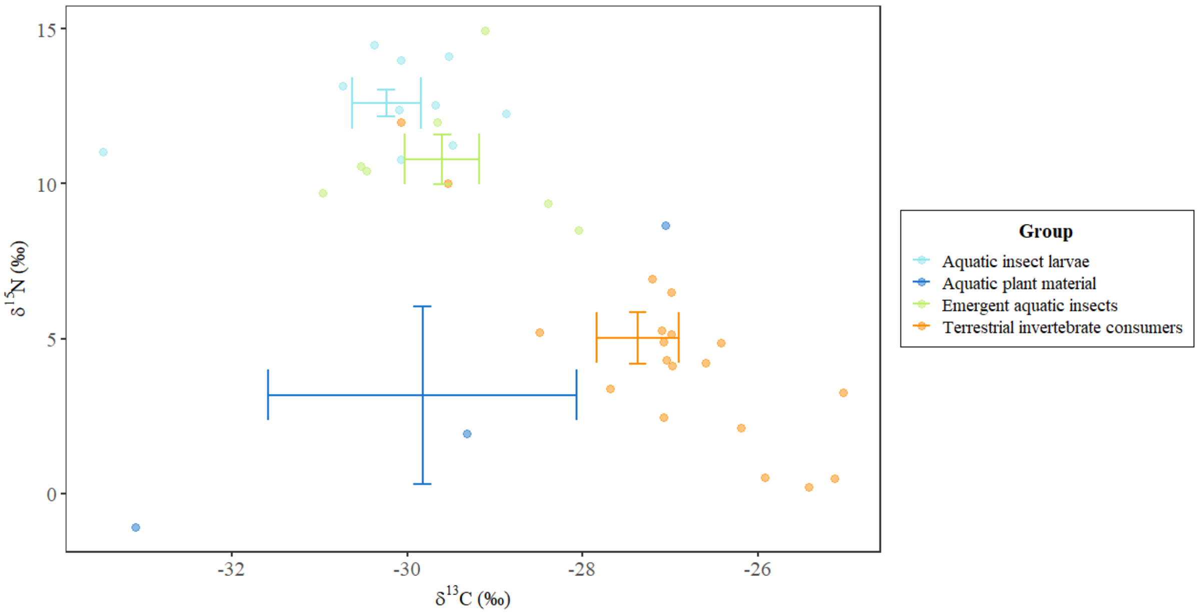

Figure S8. Values (± 1 SE) of ^{13}C vs. ^{15}N for aquatic insect larvae (light blue circles), emergent aquatic insects (green circles), aquatic plant material (dark blue circle) and terrestrial invertebrate consumers (orange circles) collected from the lake Söderhavet (KS). Two web building spider samples (orange circles) had similar $\delta^{13}C$ values as the aquatic samples.

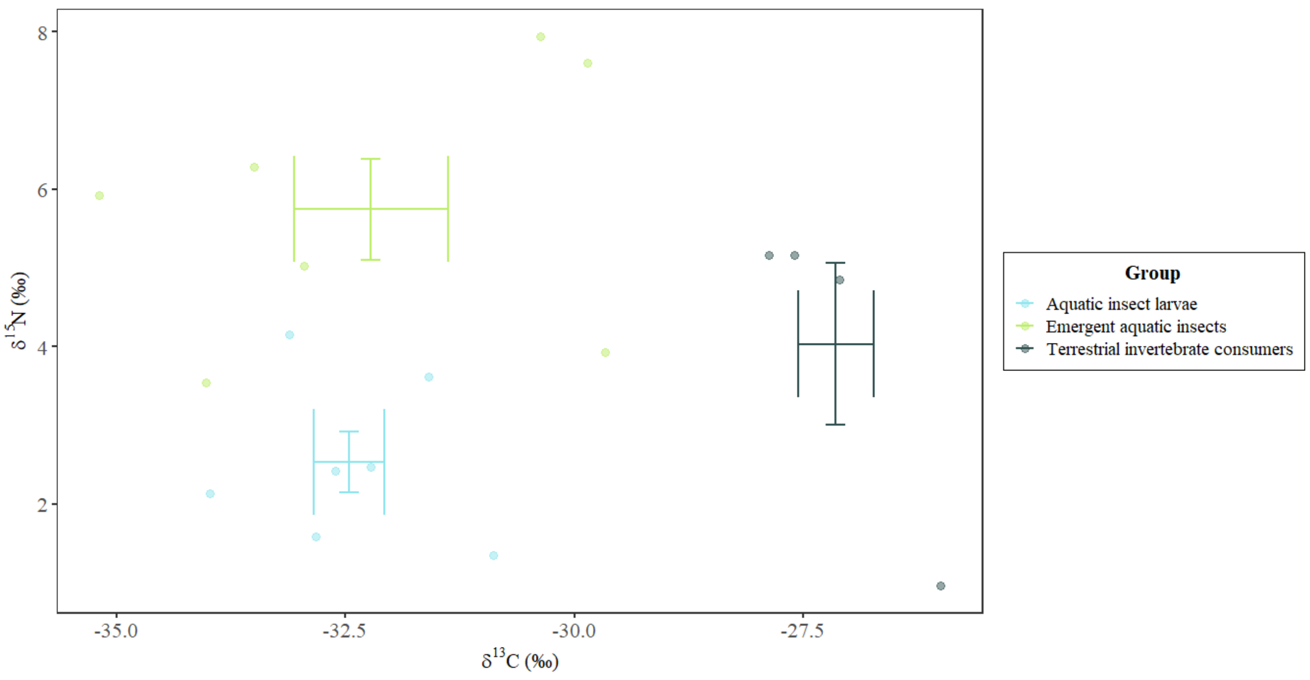

Figure S9. Values (± 1 SE) of ^{13}C vs. ^{15}N for aquatic insect larvae (light blue circles), emergent aquatic insects (green circles) and terrestrial invertebrate consumers (grey circles) collected from the reference pond (KRef).

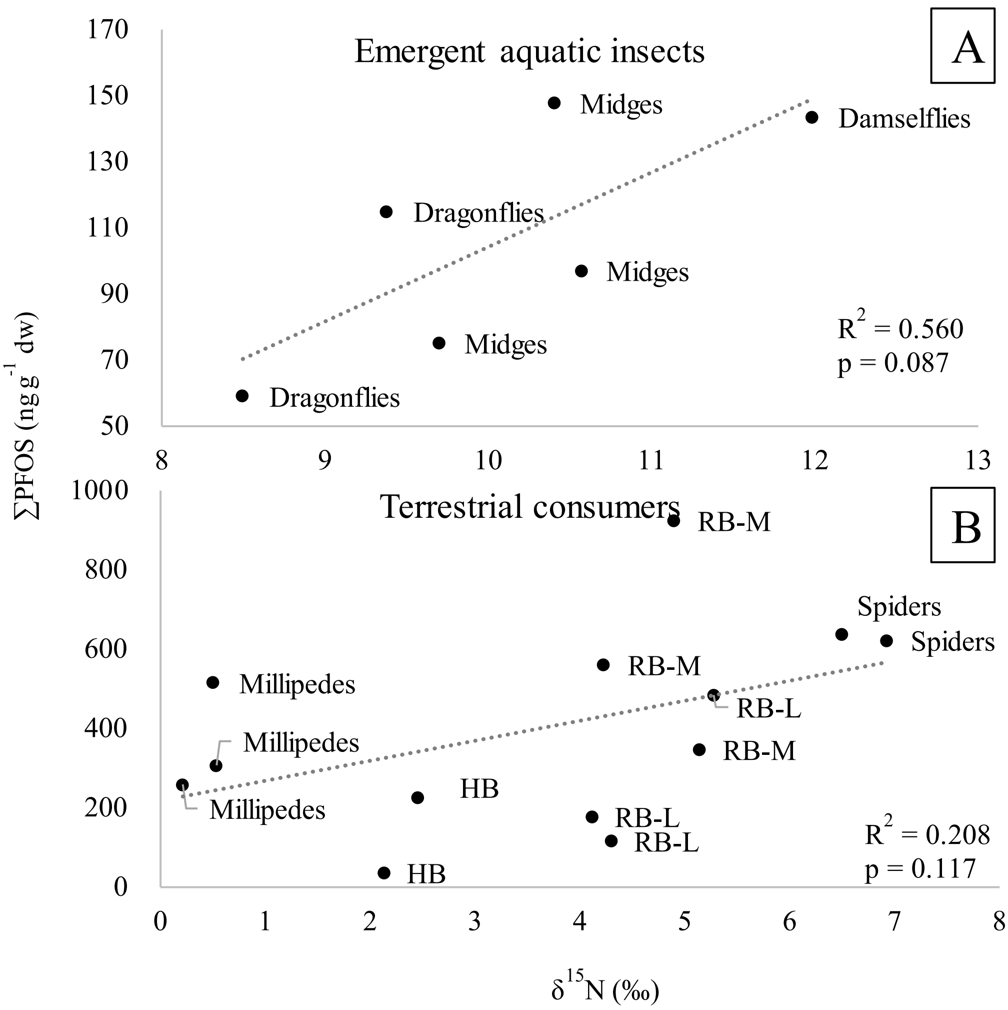

Figure S10. The sum of PFOS concentrations plotted against $\delta^{15}N$ values of emergent aquatic insects (plot A) and terrestrial consumers (plot B) from the lake (KS). Initials HB, RB-L, RB-M stand for herbivore beetles, rove beetles - large (>1.5 cm long), and rove beetles - medium (<1.5 cm long), respectively.

References

1. Cederborg, D.; Nordquist, S.; Keiter, S. Slutrapport fiskundersökning Söderhavet 2015; 1346013000; Sweco Environment AB: Karlstad, Sweden, 2016; pp 1-32.

2. Fortum Waste Solutions AB Miljökonsekvensbeskrivning Norrtorp, Kumla Kommun, Bilaga D; Sweden, 2017.

3. Stenroth, K.; Polvi, L. E.; Fältström, E.; Jonsson, M., Land-use effects on terrestrial consumers through changed size structure of aquatic insects. Freshw. Biol. 2015, 60, (1), 136-149.

4. Gobelius, L.; Hedlund, J.; Dürig, W.; Tröger, R.; Lilja, K.; Wiberg, K.; Ahrens, L., Per- and Polyfluoroalkyl Substances in Swedish Groundwater and Surface Water: Implications for Environmental Quality Standards and Drinking Water Guidelines. Environ. Sci. Technol. 2018, 52, (7), 4340-4349.

5. Powley, C. R.; George, S. W.; Ryan, T. W.; Buck, R. C., Matrix Effect-Free Analytical Methods for Determination of Perfluorinated Carboxylic Acids in Environmental Matrixes. Anal. Chem. 2005, 77, (19), 6353-6358.

6. Stenroth, K.; Polvi, L. E.; Fältström, E.; Jonsson, M., Land-use effects on terrestrial consumers through changed size structure of aquatic insects. Freshw. Biol. 2015, 60, (1), 136-149.

7. Duxbury K, Owen L, Gillingwater S, Keevil B. Naturally occurring isotopes of an analyte can interfere with doubly deuterated internal standard measurement. Ann Clin Biochem. 2008 Mar;45(Pt 2):210-2. doi: 10.1258/acb.2007.007137. PMID: 18325188.

8. Dreyer, A.; Matthias, V.; Weinberg, I.; Ebinghaus, R., Wet deposition of poly- and perfluorinated compounds in Northern Germany. Environ. Pollut. 2010, 158, (5), 1221-1227.

9. Chen, H.; Zhang, L.; Li, M.; Yao, Y.; Zhao, Z.; Munoz, G.; Sun, H., Per- and polyfluoroalkyl substances (PFASs) in precipitation from mainland China: Contributions of unknown precursors and short-chain (C2–C3) perfluoroalkyl carboxylic acids. Water Research 2019, 153.

10. Filipovic, M.; Laudon, H.; McLachlan, M. S.; Berger, U., Mass Balance of Perfluorinated Alkyl Acids in a Pristine Boreal Catchment. Environ. Sci. Technol. 2015, 49, (20), 12127-12135.

11. Johansson, J.H.; Shi, Y.; Salter, M.; Cousins, I.T. Environ. Sci.: Processes Impacts, 2018, 20, 997–1006.
